# Calcitic sclerites at base of malacostracan pleopods (Crustacea) – part of a coxa

**DOI:** 10.1186/s12862-015-0357-6

**Published:** 2015-06-19

**Authors:** Verena Kutschera, Andreas Maas, Gerd Mayer, Dieter Waloszek

**Affiliations:** Biosystematic Documentation, University of Ulm, Helmholtzstraße 20, 89081 Ulm, Germany

**Keywords:** Proximal endite, Coxa, Basipod, Pleon, Alizarin Red, Fluorescence microscopy

## Abstract

**Background:**

Cuticular specialisations such as joints and membranes play an important role in the function of arthropod limbs. This includes sclerotisations and mineral incrustations of cuticular areas to achieve either more rigidity or flexibility. The anterior eight thoracopods of Malacostraca have limb stems comprising a coxa and a basipod, which carries the two rami. Their pleopods, the limbs of the posterior trunk part, have for long been regarded to lack a coxa. Several calcitic sclerites occur in the area between ventral body and limb stem. This raises the questions: do these elements represent specialisations of the membrane due to functional requirements, and do they originate from an originally larger limb portion, i.e., the coxa, or in fact represent it.

**Results:**

We investigated 16 species of selected malacostracan taxa from all major in-groups. Calcitic sclerites occur in constant numbers and position within a species (no individual variation, and independent of specific modification such as in genital appendages). These are even constant within a supra-specific taxon, which facilitates comparisons. In general the sclerites connect via two pivot joints to the sternite medially and the tergopleura laterally, and two more to the limb stem. Based on this, we reconstructed putative ground-pattern conditions for the sclerites of the examined taxa of Malacostraca.

**Conclusions:**

The pattern of sclerites is characteristic for each monophyletic malacostracan taxon. The highest number of sclerites most likely represents the plesiomorphic state. Reduction of sclerite numbers occurs in Caridoida and its in-groups. Sclerite arrangement in these taxa provides an important character complex for phylogenetic studies. The presence of pivot joints to the body proximally and basipod distally demonstrates the existence of a coxa, which is just slightly less sclerotised, particularly on its posterior side. This can be explained by enhanced flexibility of the pleopods evolved in the course to their major role as swimming devices. Both the pivot joints and the proximal and distal extension of the calcitic sclerites demarcate the minimum area of the coxa. With this, sclerites appear as very valuable also in shedding more light on the putative relationships between Malacostraca, Myriapoda, Insecta, and Remipedia.

## Background

The membranous cuticle in the joint or articulation between body proper and appendage of sclerotised arthropods is an important structure that intermediates between rigidity and flexibility in order to facilitate the operability of an appendage. Numerous variations occur among modern arthropods ([1] and references therein for Chilopoda, Progoneata, and Hexapoda; [2] for Trilobita; [3] for Chelicerata, and crustaceans in particular [4-6]). One possibility to modify the design of arthropod joints is to stiffen parts of the membrane. These reinforcements occur in the form of sclerites within the membrane [1]. Another option is that more sclerotised cuticular elements approach each other by the formation of simple to very complex joints [7].

A well-developed basal membrane did not appear before the euarthropod level in connection with the development of a rigid, antero-posteriorly flattened stem portion, the basipod, which carried the two rami endopod and exopod [8,9]. This design permitted a more elaborate ex- and intrinsic musculature to extend from inside the body and inserting into the limb stem. Initially, the limb stem mainly moved in anterior and posterior direction. With the newly formed basal membrane, interactions could now take place between the body and the stiffer limb stem, allowing inward-outward and rotating movements. Such a "eu-arthropodium" was a multi-functional unit, with the limb stem medially armed with setae and spines to serve for food manipulation, and endopod and exopod serving more for food gathering (grabbing things) and locomotion (walking as much as swimming) ([9], see [10] for an extensive discussion of the evolution of crustacean appendages).

With few changes, this limb type was continued into the euarthropodan in-group lineages including Crustacea. This allowed the evolution as one of the crustacean novelties, a setae-bearing endite, the proximal endite, which occurred medially just below the limb stem [11-16].

In the stem species of Labrophora, the taxon that includes Phosphatocopina and Eucrustacea, the proximal endite of the anterior two postantennular appendages became modified into a distinct ring-like sclerotised proximal unit, the coxa [6,9,11,17-19].

In entomostracan crustaceans, the occurrence of a coxa is restricted to exactly those appendages, the antenna and mandible. The maxillula, the still trunk-limb shaped maxilla, and the trunk limbs retain the proximal endite. Malacostraca in this context are characterised by calcification of the cuticle, which apparently was a major trigger for the development of different life strategies within the diverse lineages.

In all malacostracans, the eight appendages of the anterior part of the two-part thorax (thoracopods 1–8 of thorax I [20]) have a divided limb stem, comprising a coxa and basipod [4,5]. However, it is still unclear if the coxa of these limbs is derived from a proximal endite or is evolved in a different way. By contrast, the six appendages of the second part of the limb-bearing trunk region of Malacostraca (thorax II [20]), the so-called pleopods or swimmerets are traditionally thought to have an undivided limb stem [21-24]. In fact, details of the limb stem are even omitted in the descriptive literature. With regard to the common occurrence of a coxa for all postantennular head limbs and those of thorax I of Malacostraca, one might expect an original coxa also on the pleopods, but which is absent, maybe it has been lost. Other scenarios are also possible. For example, a coxa originally be present on the pleopods might have fused to the limb stem [15,25-27]; or perhaps it was originally absent [8], which would reflect the phylogenetically older situation of the euarthropod condition of a limb comprising only three limb parts, i.e., even lacking the proximal endite developed in the stem lineage of Crustacea. Another alternative is the possible degeneration of either the proximal endite, or a coxa related to the loss of feeding functions.

**Figure 1:**
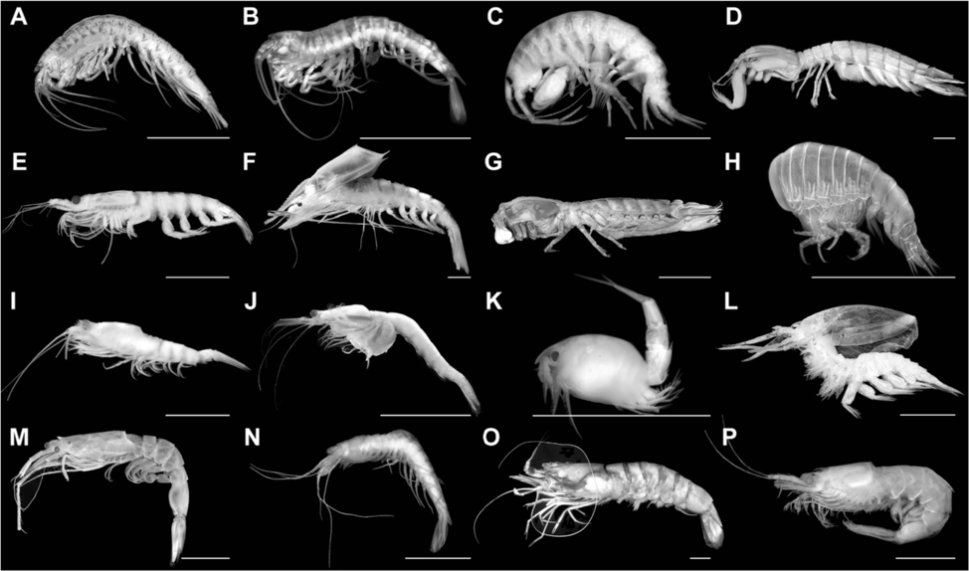
Examined species in lateral view. Scale bars 1 cm. **A**. *Anaspides tasmaniae* Thomson, 1893. **B**. *Allanaspides hickmani* Swain, Wilson & Ong, 1970. **C**. *Dikerogammarus haemobaphes* (Eichwald, 1841). **D**. *Erugosquilla massavensis* (Kossmann, 1880). **E**. *Euphausia superba* Dana, 1850. **F**. *Gnathophausia gigas* Willemoes-Suhm, 1873. **G**. *Gonodactylus chiragra* (Fabricius, 1781). **H**. *Hyperia* sp. **I**. *Lophogaster typicus* Sars, 1857. **J**. *Mysis* sp. **K**. *Nebalia bipes* (Fabricius, 1780). **L**. *Nebaliopsis* sp. **M**. *Neosergestes semissis* (Burkenroad, 1940). **N**. *Paranaspides lacustris* Smith, 1908. **O**. *Penaeus monodon* Fabricius, 1798. **P**. *Thysanopoda tricuspidata* Milne-Edwards, 1983.

**Figure 2:**
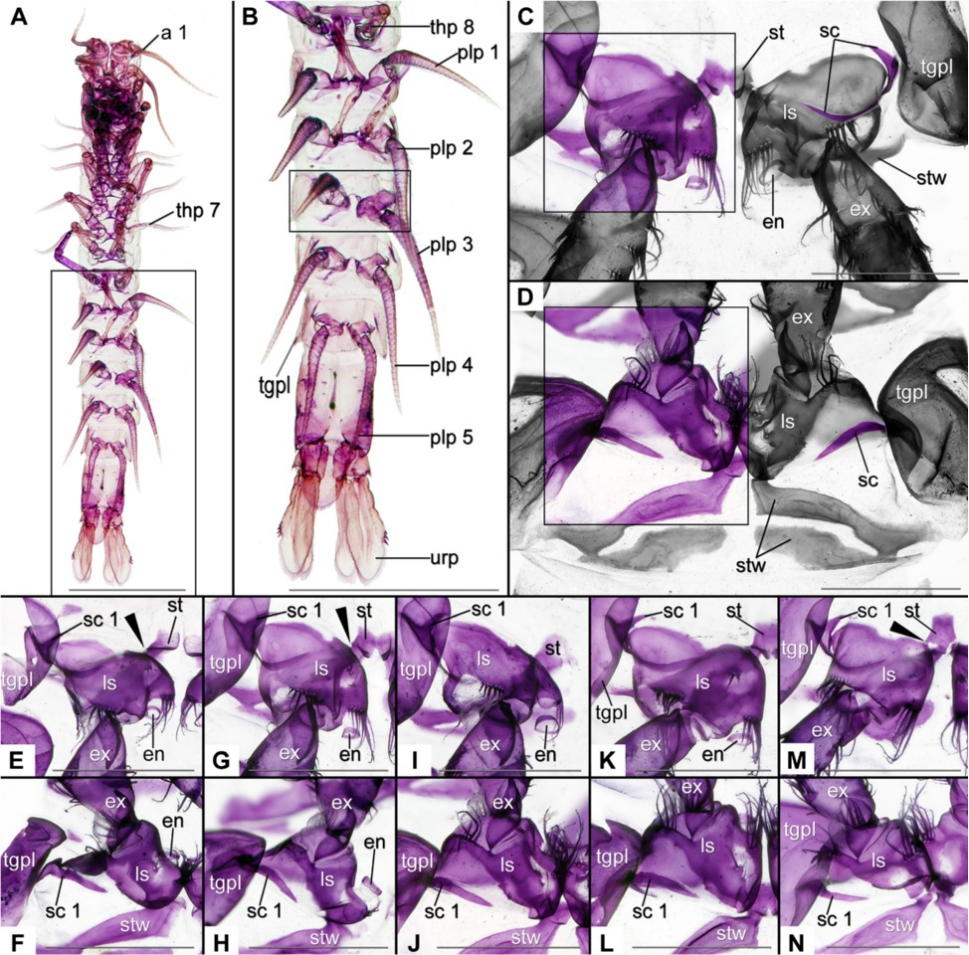
Photographs of female *Anaspides tasmaniae* Thomson, 1893 (Anaspidacea) stained with Alizarin Red. Scale bars in A & B 1 cm; C–N 1 mm. a1 = antennula; ba = basipod; en = endopod; ex = exopod; plp = pleopod; sc = sclerite; st = sternite; stw = sternitic wing; tgpl = tergopleura; thp = thoracopod; urp = uropod. **A**. Whole animal from ventral. Rectangle corresponds to area documented in B. **B**. Pleon from ventral. Rectangle illustrates documented area in C & D. **C**. Detailed view on plp articulation, here of plps 3 pointing posteriorly. Rectangle illustrates documented area in E, G, I, K, and M. Rest of image black and white except sc 1. **D**. Detailed view on articulation of plp 3 pointing anteriorly. Rectangle illustrating documented area in F, H, J, L, and N. Rest of image in black and white except sc 1. **E**. Articulation of plp 1 pointing posteriorly. Sc 1 covered by tgpl. **F**. Articulation of plp 1 pointing anteriorly with sc 1. **G**. Articulation of plp 2 pointing posteriorly with sc 1. **H**. Articulation of plp 2 pointing anteriorly with sc 1. **I**. Articulation of plp 3 pointing posteriorly with sc 1. **J**. Articulation of plp 3 with sc 1. **K**. Articulation of plp 4 pointing posteriorly with sc 1. **L**. Articulation plp 4 pointing anteriorly with sc 1. **M**. Articulation of plp 5 pointing posteriorly with sc 1. **N**. Articulation of plp 5 pointing anteriorly with sc 1.

**Figure 3:**
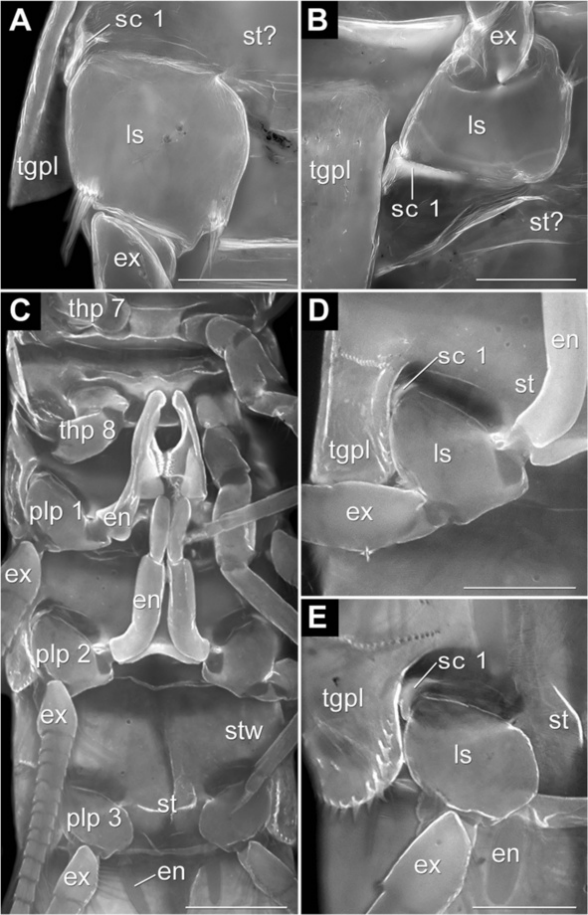
Fluorescence micrographs of Anaspidacea. A–B female *Allanaspides hickmani* Swain, Wilson & Ong, 1970 and C–E male *Paranaspides lacustris* Smith, 1908. Scale bars A & B 0.5 mm; C–E 1 mm. Abbreviations as before. **A**. Detailed view on articulation of plp 3 pointing posteriorly. Note sc 1. **B**. Articulation membrane of plp 3 pointing anteriorly with sc 1. **C**. Ventral side displaying plp 1 & 2 with en modified to petasma. **D**. Detailed view on articulation area of plp 2 pointing posteriorly and with modified en. Note sc 1. **E**. View on articulation of plp 3 pointing posteriorly with sc 1.

**Figure 4:**
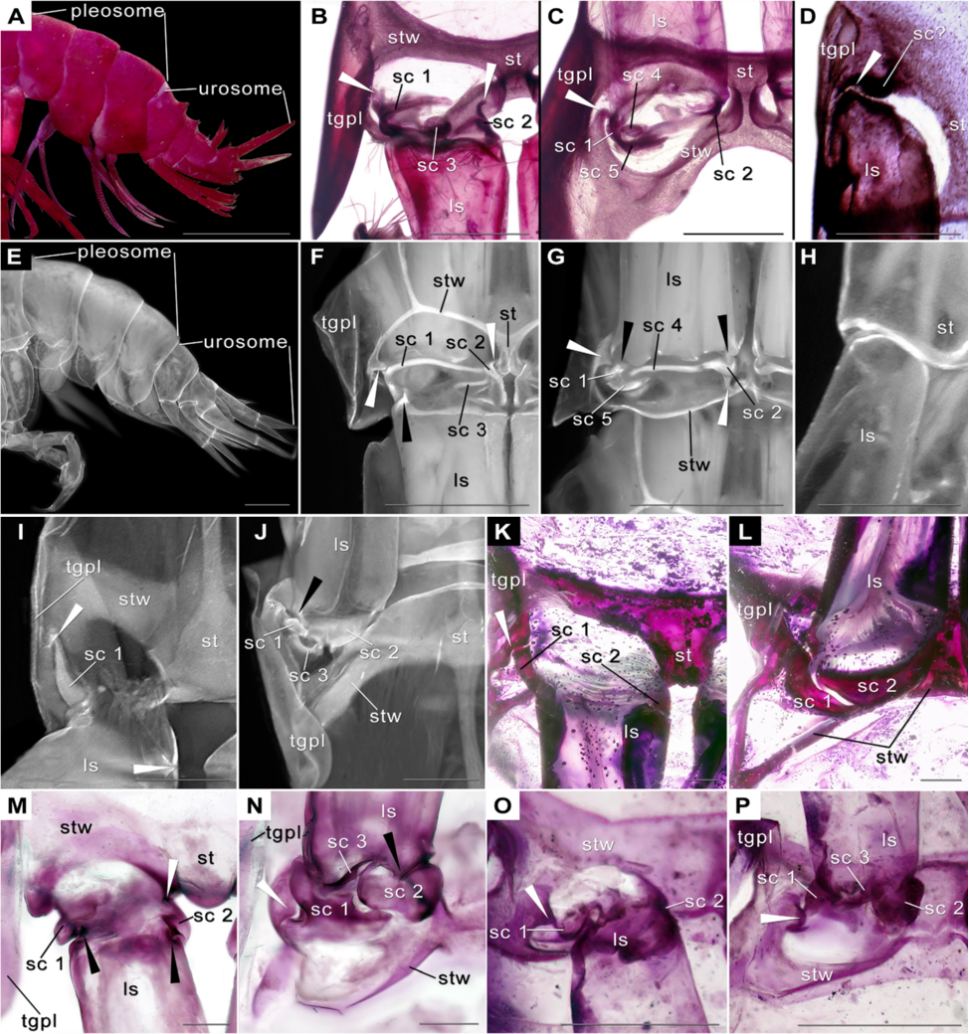
Pleon details of malacostracan representatives. A–D *Dikerogammarus haemobaphes* (Eichwald, 1841) (Amphipoda). E–H *Hyperia* sp. (Amphipoda). I–J Fluorescence micrographs of *Neosergestes semissis* (Burkenroad, 1940) (Dendrobranchiata). K–L *Penaeus monodon* Fabricius, 1798 (Dendrobranchiata). M–N *Euphausia superba* Dana, 1850 (Euphausiacea). O–P *Thysanopoda tricuspidata* Milne-Edwards, 1983. A–D, K–P Photographs of Alizarin-Red stained specimens. E–J fluorescence micrographs. Scale bars A = 1 cm; B–P = 1 mm. Abbreviations as before. **A**. Pleon from lateral displaying tagmatisation. **B**. Articulation of plp 3 pointing posteriorly with sc 1– 3. White arrowhead = joint tergopleura–sc 1. **C**. Articulation of plp 3 pointing anteriorly with scl 1–2, 4–6. White arrowhead as in B. **D**. Articulation of plp 5 pointing posteriorly. White arrowhead = joint. Note darker area anterior to basipod. **E**. Pleon of *Hyperia* sp. from lateral displaying tagmatisation. **F**. Articulation of plp 3 pointing posteriorly with sc 1–3. Tgpl slightly deformed. White arrowheads = joints of tgpl–sc 1, st–sc 2. Black arrowhead = joints of ba–sc 1. **G**. Articulation on plp 3 pointing anteriorly with sc 1–2, 4–6. Arrowheads as in F. **H**. Articulation of plp 5 pointing posteriorly. **I**. Articulation of plp 3 pointing posteriorly with sc 1 anchored deeply in tergopleura (white arrowhead). **J**. Articulation of plp 3 pointing anteriorly with sc 1–3. Note uparching sternite on lateral side marked by white star. Black arrowhead = joint ba–sc 1. **K**. Articulation of plp 3 pointing posteriorly with sc 1, 2. White arrowhead = joint tgpl–sc 1. **L**. Articulation of plp 3 pointing anteriorly with sc 1–2. Tgpl removed. **M**. Articulation of plp 3 pointing posteriorly with sc 1–2. White arrowhead = joint st–sc 2. **N**. Articulation of plp 3 pointing anteriorly with sc 1–3. White arrowhead = joint tgpl–sc 1. Black arrowhead = joint ba–sc 2. **O**. Articulation of plp 3 pointing posteriorly with sc 1–2. White arrowhead = joint tgpl–sc 1. **P**. Articulation of plp 3 pointing anteriorly with sc 1–3. White arrowhead as in O.

**Figure 5:**
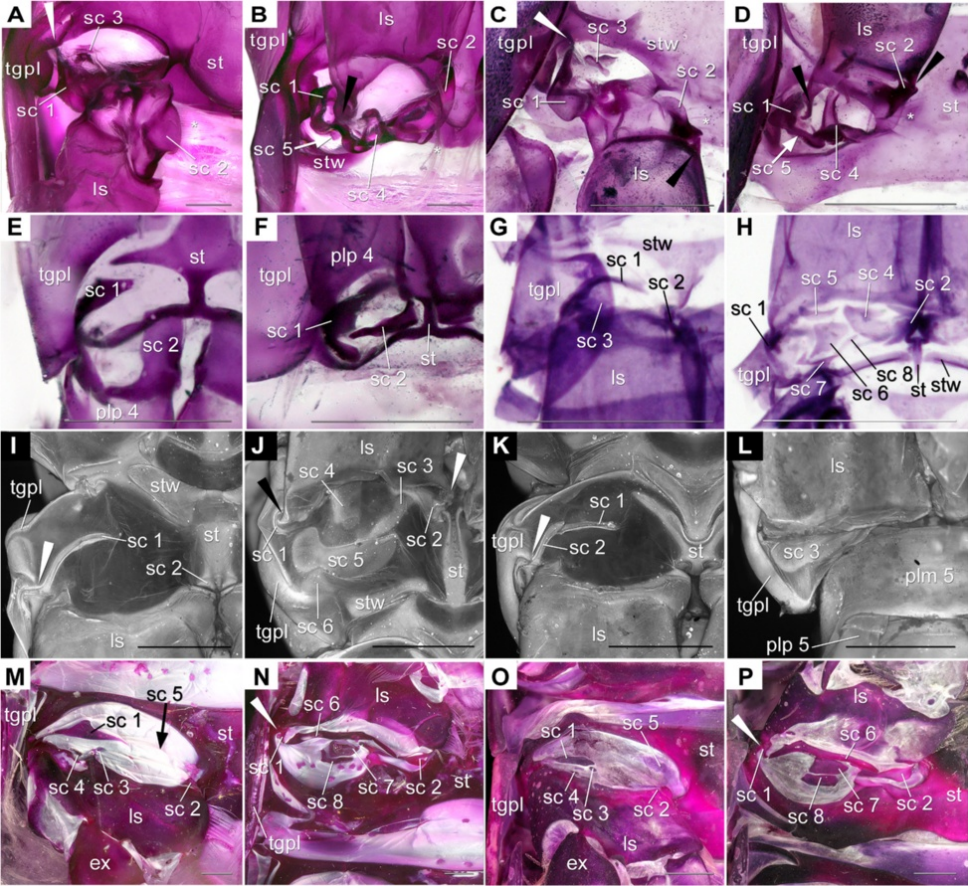
Pleopod articulations of different Malacostraca. A–B *Lophogaster typicus* Sars, 1857 (Lophogastrida). C–D *Neognathophausia gigas* (Willemoes-Suhm, 1875) (Lophogastrida). E–F *Mysis* sp. (Mysida), male. G–H *Nebalia bipes* (Fabricius, 1780) (Phyllocarida). I–L *Nebaliopsis* sp. (Phyllocarida). M–N *Erugosquilla massavensis* (Kossmann, 1880) (Hoplocarida) O–P *Gonodactylus chiragra* (Fabricius, 1781) (Hoplocarida). A–H, M–P Photographs of Alizarin-Red stained specimens. I–L fluorescence micrographs. Scale bars 1 mm. Abbreviations as before. **A**. Plp 3 pointing posteriorly with sc 1– 3. White arrowhead = joint tgpl–sc 1. White star = setae at sc 2. **B**. Plp 3 pointing anteriorly with sc 1–2, 4. Black arrowhead = joint ba–sc 1. White arrow marks position sc 5. White stars = setae at sc 2. **C**. Plp 3 pointing posteriorly with sc 1–3. White arrowhead = joint of tgpl–sc 1. White star = setae at sc 2. Black arrowhead = joint ba–sc 2. **D**. Plp 3 pointing anteriorly with sc 1–2, 4. Black arrowheads = joints ba-sc 1, ba-sc 2. White arrow marks position sc 5. White star = setae at sc 2. **E**. Plp 4 pointing posteriorly with sc 1–2. **F**. Plp 4 pointing anteriorly with sc 1–2. **G**. Plp 3 pointing posteriorly with sc 1– 3. Sc 1 overlapped by tgpl. **H**. Plp 3 pointing anteriorly with sc 1–2, 4–8. **I**. Plp 3 pointing posteriorly with sc 1–2. White arrowhead = joint tgpl–sc 1. **J**. Plp 3 pointing anteriorly with sc 1–6. White arrowheads = joints ba–sc 1, ba–sc 2. **K**. Plp 4 pointing posteriorly with sc 1–2. white arrowhead = joint tgpl–sc 2. **L**. Plp 4 pointing anteriorly with sc 3. **M**. Plp 3 pointing posteriorly with sc 1–4. Position of sclerite 5 marked by black arrow. **N**. Plp 3 pointing anteriorly with sc 1–2, 6–8. White arrowhead = joint tgpl-sc 1. **O**. Plp 3 pointing posteriorly with sc 1–5. **P**. Plp 3 pointing anteriorly with sc 1–2, 6–8. White arrowhead = joint tgpl–sc 1.

**Figure 6:**
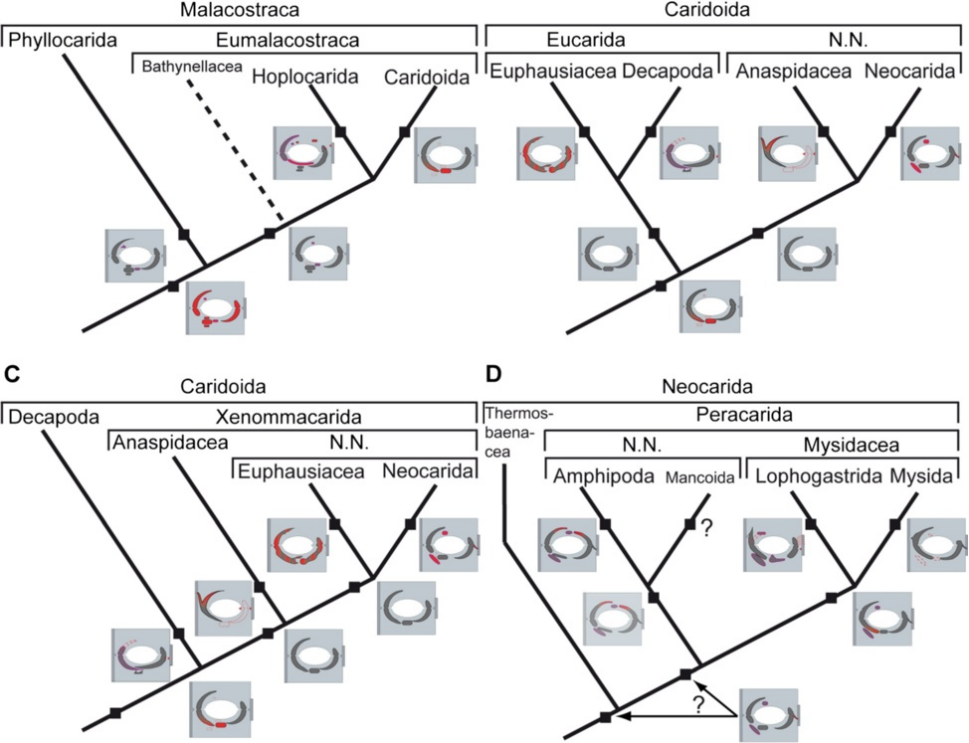
Mapping and ground-pattern reconstructions. A–B, D refer to phylogeny proposed by [35]. C refers to phylogeny proposed by [36]. Mapped are our findings (Table 2). Ground pattern states for higher taxa were reconstructed accordingly. Red = possible autapomorphic states. Purple = sclerites with apodemes. Note that Decapoda here is short term of Decapoda sensu lato. **A**. Relationships of basal Malacostraca. **B**. Phylogeny of Caridoida after [35]. **C.** Phylogeny of Caridoida after [36]. **D.** Relationships in Neocarida. See also [42], also for the putative position of Bathynellacea.

In fact, the pleon is a functionally highly important body tagma of malacostracan crustaceans, e.g., for swimming, caridoid escape reaction, brood care, but the pleon has been strangely little considered so far for phylogenetic purposes, except [28] who demonstrated the potential of the pleon and its morphological details for phylogeny analyses and demanded for more data to be collected, compared and interpreted. Therefore, it seemed of high relevance for us to study the details of the pleon morphology with particular regard to the basal joint and its structures associated with it. We started to particularly search for structures that might hint at the original situation at the base of the pleopods of malacostracans. Our goal was to find hints for the original presence of either the "proximal endite", or a "coxa". We examined what appears to be the exclusively membranous area extending between the ventral body surface and the comprehensive pleopod limb stem ("the pleopod-body articulation") in a wide array of malacostracan taxa with Alizarin-Red staining (which highlights calcified areas) and fluorescence microscopy (Figures 1, 2, 3, 4 and 5; Table 1). We detected several small, calcified areas (termed "sclerites" herein) within the pleopod-body articulation (Figures 2, 3 and 4; Table 1). Considering up to eight individuals for each species and similar patterns in closely related species indicate that the patterns presented here are real and not artefacts produced either by fixation methods, or by the time lag to the next moult. We also used normal light and scanning electron microscopy to check the proximal limb area for its structural composition. Besides discussion of the possible role of the sclerites in the functionality of the articulations, we will also evaluate their potential for phylogenetic considerations (Figure 6; Table 2 and Table 3) and provide a suggestion about the morphology of the pleopods.

**Table 1:** Summarising table containing main results of sclerite examination

**Species**	**plp**	**Number**	**Scheme of sclerites**	**Pivot joints**		**apo**
				**Laterally**	**Medially**	
*Anaspides tasmaniae* Thomson, 1893	1–5	1		tgpl-sc 1	st-ba	-
				sc 1-ba		
*Allanaspides hickmani* Swain, Wilson & Ong, 1970	1–5	1		tgpl-sc 1	st-ba	?
				sc 1-ba		
*Dikerogammarus haemobaphes* (Eichwald, 1841)	1–3	5		tgpl-sc 1	st-sc 2	sc 3
				sc 1-ba	sc 2-ba	sc 5
*Erugosquilla massavensis* (Kossmann, 1880)	1–5	8		tgpl-sc 1	st-sc 2	sc 1
				sc 1-ba	sc 2-ba	sc 4
						sc 6
						sc 8
*Euphausia superba* Dana, 1850	1–5	3		tgpl-sc 1	st-sc 2	-
				sc 1-ba	sc 2-ba	
*Gnathophausia gigas* Willemoes-Suhm, 1873	1–5	5		tgpl-sc 1	st-sc 2	sc 3
				sc 1-ba	sc 2-ba	sc 4
						sc 5
*Gonodactylus chiragra* (Fabricius, 1781)	1–5	8		tgpl-sc 1	st-sc2	sc1
				sc1-ba	sc2-ba	sc4
						sc6
*Hyperia* sp.	1–3	5		tgpl-sc 1	st-sc 2	?
				sc 1-ba	sc 2-ba	
*Lophogaster typicus* Sars, 1857	1–5	5		tgpl-sc 1	st-sc 2	sc 3
				sc 1-ba	sc 2-ba	sc 4
						sc 5
*Mysis* sp.	1–5	2		tgpl-sc 1	st-sc 2	-
				sc 1-ba	sc 2-ba	
*Nebalia bipes* (Fabricius, 1780)	1–3	8		tgpl-sc 1	st-sc 2	sc 3
				sc 1-ba	sc 2-ba	sc 4
*Nebaliopsis* sp.	1–3	6		tgpl-sc 1	st-sc 2	?
				sc 1-ba	sc 2-ba	
*Neosergestes semissis* (Burkenroad, 1940)	1–5	3		tgpl-sc 1	st-sc 2	sc 1
				sc 1-ba	sc 2-ba	
*Paranaspides lacustris* Smith, 1908	1–5	1		tgpl-sc 1	st-ba	?
				sc 1-ba		
*Penaeus monodon* Fabricius, 1798	1–5	2		tgpl-sc 1	st-sc 2	-
				sc 1-ba	sc 2-ba	
*Thysanopoda tricuspidata* Milne-Edwards, 1837	1–5	3		tgpl-sc 1	st-sc 2	-
				sc 1-ba	sc 2-ba	

Sclerites with apodemes with purple line. Abbreviations: *apo* apodemes, *ba* basipod, *n* number of scerites, *plp* given description valid for pleopods, *sc* sclerite, *st* sternite, *tgpl* tergopleura. *) scheme for large sclerites found on plp 3, 4 of males of *Mysis* sp.

**Table 2:** Ground-pattern situations reconstructed for the examined taxa

**Taxon**	**scl gp**	**n**	**apo**	**lat art**	**disloc**	**med art**	**disloc**
Anaspidacea		1	no	no	–	ba – st	no
Decapoda		3	1	ba – scl – tgpl	no	ba – scl – st	post
Euphausiacea		3	no	ba – scl – tgpl	no	ba – scl – st	no
Mysida		2	no	ba – scl – tgpl	ant	ba – scl + ext – st	no
Amphipoda		5	2	ba – scl – tgpl	no	ba – scl + ext – st	no
Lophogastrida		6	3	ba – scl – tgpl	ant	ba – scl – st	post
Stomatopoda		8	3	ba – scl – tgpl	no	ba – scl – st	ant
Phyllocarida		7	2	ba – scl – tgpl	no	ba – scl – st	no

Schemes illustrate articulation membrane. Purple outline for a sclerites with apodemes. Abbreviations other than in Table 1: disloc, dislocation; gp, ground pattern; mart, median articulation; lat art, lateral articulation; med art, median articulation.

**Table 3:** Symplesiomorphic conditions of sclerites

	**Phyllocarida**		**Hoplocarida**			**Euphausiacea**	**Decapoda**	**Anaspidacea**			**Amphipoda**	**Mysida**	**Lophogastrida**
Malacostraca	*Nebalia bipes* (Fabricius, 1780)	*Nebaliopsis* sp.	Eumalacostraca	*Erugosquilla massavensis* (Kossmann, 1880) *Gonodactylus chiragra* (Fabricius, 1781)	Caridoida	*Euphausia superba* (Dana, 1850) *Thysanopoda tricuspidata* Milne-Edwards, 1837	*Neosergestes semissis* (Burkenroad, 1940)	*Penaeus monodon* Fabricius, 1798	*Anaspides tasmaniae* Thomson, 1893 *Allanaspides hickmani* Swain, Ong & Wilson, 1970 *Paranaspides lacustris* Smith, 1908	Peracarida	*Dikerogammarus haemobaphes* (Eichwald, 1841) *Hyperia* sp.	*Mysis* sp.	*Gnathophausia ingens* Willemoes-Suhm, 1873 *Lophogaster typicus* Sars, 1857
la sc	sc 1	sc 1	la sc	sc 1*	la sc	sc 1	sc 1*	sc 1	sc 1	la sc	sc 1	sc 1	sc 1
me sc	sc 2	sc 2	me sc	sc 2	me sc	sc 2	sc 2	sc 2	-	me sc	sc 2	sc 2	sc 2
an sc*	sc 3*	-	an sc*	sc 3*	an sc*	-	-	-	-	an sc*	sc 3*	-	sc 3*
po-di sc	sc 5	sc 4	-	-	-	-	-	-	-	-	-	-	-
po-me sc*	sc 4*	sc 3	pome sc*	sc 6*	pome sc?	sc 3?	sc 3?	-	-	po-di sc?	sc 4	-	sc 4
po-ce sc	sc 6	sc 5	poce sc	sc 7	poce?	sc 3?	sc 3?	-	-	po-pr sc?	sc 5*	-	sc 5*
po-pr sc	sc 7	sc 6	poce sc	sc 8* (*E.m.)*	-?	-	-	-	-	-	-	-	-

Left column malacostracan ground pattern. Corresponding sclerites given in right columns for each taxon/ species. Abbreviations: an anterior, ce central, di distal, (E.m.) Apodeme only in Erugosquilla massavensis Kossmann 1880, la lateral, me median, po posterior, pr proximal, sc sclerite, ? = uncertain homologisation; * = sclerite with apodeme.

**Table 4:** Species examined, taxon affiliation, and applied methods for examination of pleopod-body articulation

**Species**	**Taxon**	**Reg**	**AR**	**Mph**	**FM**	**Di**
*Anaspides tasmaniae* Thomson, 1893	Caridoida Hessler, 1982 Anaspidacea Calman, 1904	**-**	+	+	**-**	+
*Allanaspides hickmani* Swain, Wilson & Ong, 1970	Caridoida Hessler, 1982 Anaspidacea Calman, 1904	**-**	**-**	**-**	+	**-**
*Dikerogammarus haemobaphes* (Eichwald, 1841)	Caridoida Hessler, 1982 Peracarida Calman, 1904 Amphipoda, Gammaridea	**-**	+	+	**-**	+
*Erugosquilla massavensis* (Kossmann, 1880)	Hoplocarida Calman, 1904 Stomatopoda Latreille, 1817	**-**	+	+	**-**	**-**
*Euphausia superba* Dana, 1850	Caridoida Hessler, 1982 Eucarida Calman, 1904 Euphausiacea Dana, 1852	**-**	+	+	**-**	+
*Gnathophausia gigas* Willemoes-Suhm, 1873	Caridoida Hessler, 1982 Peracarida Calman, 1904 Lophogastrida Sars, 1870	**-**	+	+	**-**	+
*Gonodactylus chiragra* (Fabricius, 1781)	Hoplocarida Calman, 1904 Stomatopoda Latreille, 1817	+	+	+	**-**	+
*Hyperia* sp.	Caridoida Hessler, 1982 Peracarida Calman, 1904 Amphipoda, Hyperiidae	**-**	**-**	-	+	**-**
*Lophogaster typicus* Sars, 1857	Caridoida Hessler, 1982 Peracarida Calman, 1904 Lophogastrida Sars, 1870	**-**	+	+	**-**	+
*Mysis* sp.	Caridoida Hessler, 1982 Peracarida Calman, 1904 Mysida Boas, 1883	**-**	+	+	**-**	+
*Nebalia bipes* (Fabricius, 1780)	Phyllocarida Packard, 1879 Leptostraca Claus, 1880	**-**	+	+	**-**	
*Nebaliopsis* sp.	Phyllocarida Packard, 1879 Leptostraca Claus, 1880	**-**	**-**	**-**	+	-
*Neosergestes semissis* (Burkenroad, 1940)	Caridoida Hessler, 1982 Eucarida Calman, 1904 Decapoda Latreille, 1802 Dendrobranchiata Bate, 1888	**-**	**-**	**-**	+	+
*Paranaspides lacustris* Smith, 1908	Caridoida Hessler, 1982 Anaspidacea Calman, 1904	**-**	**-**	**-**	+	-
*Penaeus monodon* Fabricius, 1798	Caridoida Hessler, 1982 Eucarida Calman, 1904 Decapoda Latreille, 1802 Dendrobranchiata Bate, 1888	**-**	+	+	**-**	+
*Thysanopoda tricuspidata* Milne-Edwards, 1837	Caridoida Hessler, 1982 Eucarida Calman, 1904 Euphausiacea Dana, 1852	**-**	+	+	**-**	+

Abbreviations: *AR* Staining with Alizarin Red, *Di* Dissection, *FM* Fluorescence microscopy, *Mph* Macrophotography, *Reg* Regeneration in NaCl.

## Results

Details revealed on the ventral side of a pleomere and its body–limb articulations include the shape of tergopleurae, sternites, and insertion of limbs, as well as the number, shape, and position of the sclerites within the arthrodial membranes. In all species we examined, the morphology is generally similar for the first to fifth pleomeres (4 in *Nebalia bipes* (Fabricius, 1780) and *Nebaliopsis* sp.) (Figure 1). Therefore, we describe the situation in a general way for each species, applicable to pleomeres 1–5 (1–4) (see below, Table 1). Additionally, we indicate sexual dimorphism occurring in some of these species, including the sternites, and also record exceptions we uncovered from what one can read in generalised descriptions.

### Anaspidacea (Figures 2 and 3)

#### *Anaspides tasmaniae* Thomson, 1893 (Figures 1A, 2A–N; Table 4)

Calcified tergopleura (tgpl) borders pleomere (plm) laterally (Figure 2A–D); calcified, rhombic plate medially interpreted as sternite (st) (Figure 2C, E, G, I, K, M); st subdivided at plm1 (Figure 2E), undivided plm2–5 (Figure G, I, K, M); st drawn out posteriorly into sternitic wings (stw) (Figure 2D).

Limb stem (ls) truncated cone inserting ventrally at plm (Figure 2A–N); carries multi-annulated, distally-tapering exopod (ex) (Figure 2A, B) and comparably small, cylindrical endopod (en) (Figure 2E, G, I, K, M); pleopod (plp) 5 without en (Figure 2M); postero-laterally, ls proximally excavated (Figure 2F, H, J, L, N).

A single semicircular sclerite lies laterally within the membrane between ls and tgpl (Figure 2C–N; Table 1, also for more details). The sclerite is anteriorly bifurcate; at the split of the fork, the ls articulates with this sclerite 1 laterally. Medially, the ls articulates directly with the st via a pivot joint (Figure 2E, G, M; black arrowheads; Table 1). The median half of the bifurcated end of sclerite 1 encompasses the ls anteriorly while the lateral half articulates with the tgpl via a pivot joint. No apodemes arise from the inside of this sclerite.

Sexual dimorphism: plp 1–2 modified into petasma in males, yet both with single lateral, anteriorly bifurcate sclerite forming joints with ls and tgpl.

#### *Allanaspides helonomus* Swain, Wilson & Ong, 1970 **(Figures ****1**B, 3**A–B,** Table 4)

Tgpl similar to *An. tasmaniae* (Figure 3A); st barely calcified thus weakly fluorescent and barely visible; ls truncated cone inserting ventrally at the pleomere (Figure 3A); ls carrying multi-annulated, distally tapering ex (Figure 3A–B); no en at plp 1–5.

A single sclerite is present laterally in the articulation membrane of *Al. helonomus* (Figure 3A–B; Table 3). The sclerite is semicircular and anteriorly bifurcate (Figure 3A–B). The median half of the bifurcated end encompasses the ls anteriorly and forms a joint with it, while the lateral half articulates with the tgpl via a pivot joint (Table 1). Medially, the ls articulates directly with the st via a pivot joint. Examination of internal apodemes was not possible (Table 1 and Table 3). Differences between the sexes were not recognised.

#### *Paranaspides lacustris* Smith, 1908 **(Figures** **1**N, 3C–E, Table 4)

Calcified tgpl borders pleomere laterally (Figure 3D–E); divided, median plate interpreted as st with stw extending antero-laterally; plp insert postero-laterally (Figure 3C–E); ls truncated cone inserting latero-ventrally (Figure 3C–E); ls carries multi-annulated, distally tapering ex (Figure 3C) and comparably small, lentiform en (Figure 3C, E); exceptions are plp1–2 in males forming petasm with en (Figure 3C–D); ls overlaps st medio-proximally.

A single sclerite is present laterally in the articulation membrane of *Pa. lacustris* (Figure 3D–E; Table 1). The sclerite is semicircular and anteriorly bifurcate and is located directly between ls and tgpl (Figure 3D–E). It forms articulations with both the tgpl and the ls (Table 1). We could not determine the presence of internal apodemes (Table 1). Differences between the sexes were not recognised.

### Amphipoda (Figure **4**A–H)

The pleon of Amphipoda is divided into a pleosome and urosome (Figure 4A, E) [32]. Accordingly, the pleopod articulations show different morphologies and are described separately. Although the sixth pleopod is part of the urosome, it is omitted here.

#### *Dikerogammarus haemobaphes* (Eichwald, 1841) **(Figures** **1**C, 4**A–D**, Table 4)

*Pleosome. –* Calcified tgpl borders pleomere laterally (Figure 4B–C); st, anterior and posterior stw strongly calcified (Figure 4B–C); stw merge into tgpl laterally (Figure 4B–C); ls sub-cylindrical, inserts on pleomere ventrally (Figure 4B–C); rami multi-annulated, ls drawn out proximo-medially and proximo-laterally into knobs forming pivot joints.

There are five sclerites in the articulation membrane of the pleopods (Figure 4B–C; Table 1). Sclerite 1 lies laterally in the articulation membrane, is semicircular and forms a bifurcated fork anteriorly. The latero-proximal process of the fork forms a pivot joint with the knob of the fusion area of tgpl and stw (Figure 4B; white arrowhead; Table 1). Distally, sclerite 1 forms a pivot joint with the ls (Table 1). Sclerite 2 lies on the median side (Figure 4B–C). It is semicircular and has a proximal outgrowth that forms a joint with the st (Figure 4B; white arrowhead). Distally, sclerite 2 articulates with the ls. A third sclerite is sited on the anterior side of the articulation membrane between sclerites 1 and 2 (Figure 4B). It is round and lies directly proximal to the ls. On the posterior side two additional sclerites are found (Figure 4C): sclerite 4 is oval and lies median to sclerite 1; sclerite 5 is located directly proximal to sclerite 1 and 4. The antero-lateral half of the sub-rectangular, elongated sclerite 5 contacts the postero-median margin of sclerite 1. Sclerite 3 and 5 possess apodemes.

*Urosome. –* The morphology of the urosomal segments differs from that of the pleosomal ones enormously. The calcified ventral areas are sub-rectangular with two oval holes posteriorly (Figure 4D). Thus the posterior stw is very slender compared to the anterior wing; ls is cylindrical, carrying non-annulated rami, inserting latero-ventrally at urosomal segment (Figure 4D).

No sclerites are present in the articulation membranes of pleopods 4 and 5. However, there is a strongly calcified area antero-laterally at the pleopod insertion. This area forms a pivot joint with the limb stem (Figure 4D; white arrowhead). Speculatively, the strong calcification and the joint might be hints that a sclerite is fused to the sternitic wing. No differences between the two sexes of *D. haemobaphes* could be recognised.

#### *Hyperia* sp. (Figures **1**H, 4E–H, Table 4)

*Pleosome. –* Calcified tgpl borders pleomere laterally (Figure 4F); st and stw relatively slender (Figure 4F–G); stw merge into tgpl laterally (Figure 4F–G); ls sub-cylindrical (Figure 4F–G), carrying multi-annulated rami; ls drawn out proximo-medially and proximo-laterally into knobs forming pivot joints (Figure 4F–G; black arrowheads).

There are five sclerites in the articulation membrane of pleopods 1–3 of the pleosomal segments (Figure 4F–G; Table 1). Sclerite 1 lies laterally in the articulation membrane, is semicircular on the lateral side, and extends rather straight, latero-medially orientated at the anterior side of the articulation membrane (Figure 4F). Its latero-proximal part forms an articulation with the tgpl (Figure 4F–G; white arrowheads; Table 1). Sclerite 1 forms a pivot joint with the ls latero-distally (Figure 4G; black arrowhead; Table 1). Sclerite 2 lies medially (Figure 4F–G). It is semi-circular and has a proximal outgrowth that contacts the st and forms a pivot joint with it (Figure 4F–G; white arrowhead; Table 1). Sclerite 2 forms a pivot joint with the ls postero-medially (Figure 4G, black arrowhead; Table 1). Sclerite 1 and 2 contact each other antero-medially (Figure 4F). A third elongate sclerite 3 lies distal to this connection (Figure 4F). It is crowbar-shaped, latero-medially orientated, and contacts sclerite 2 with its median side distally (Figure 4F). On the posterior side two other sclerites are present (Figure 4G): sclerite 4 is elongated, latero-medially orientated, and lies centrally, close to the ls in the articulation membrane contacting sclerite 1 laterally and sclerite 2 medially. The crescentic sclerite 5 lies laterally, proximal to the connection of sclerite 1 and 4. Whether internal apodemes are present could not be examined (Table 1).

*Urosome. –* The urosomal segments do not have as distinctive tgpl as the pleosomal segments. The tgpl merge smoothly with the st. Nevertheless, the st seems to be sub-rectangular with two semicircular, postero-lateral recesses. At these recesses the plp insert at the latero-posterior margin of a urosomal segment (Figure 4E, H). The ls is cylindrical and carries two non-annulated portions, endopod and exopod. No sclerites are present in the articulation membranes of pleopods 4 and 5*.* No differences have been recognised between the two sexes*.*

### Decapoda (Figure **4**I–L)

#### *Neosergestes semissis* (Burkenroad, 1940) (Figures **1**M, 4I–J; Table 4)

The calcified tgpl contacts ventral surface of pleomere laterally; st and stw strongly calcified (Figure 4I–J); stw merge into tgpl laterally (Figure 4F–G ls cylindrical and thickened in proximo-distal mid-section; ls carrying annulated rami; ls drawn out proximo-medially and proximo-laterally into knobs forming pivot joints (Figure 4J; black arrowhead).

Three sclerites are present (Figure 4I–J; Table 1). Sclerite 1 lies laterally and is semicircular with a tipped anterior end (Figure 4I). This tipped end is anchored in the tgpl (Figure 4 I; white arrowhead). The lateral side arches upward resulting in a fold when the pleopod is bent anteriorly (Figure 4J; white star). The distal margin of sclerite 1 forms a pivot joint with the ls posteriorly (Figure 4J; black arrowhead; Table 1). Sclerite 2 lies posteriorly and is crescentic (Figure 4J). Medio-proximally it forms a pivot joint with the st, medio-distally with the ls (Table 1). Sclerite 2 contacts sclerite 1 on the posterior side of the articulation membrane (Figure 4J). Sclerite 3 is C-shaped and lies proximal to the connection of sclerite 1 and sclerite 2 (Figure 4J). The tipped, anterior end of sclerite 1 functions as an apodeme (Table 1). No other apodemes are present (Table 1). Only females were available for study, thus we cannot offer any data for the males of *Neo. semissis*.

#### *Penaeus monodon* Fabricius, 1798 (Figures **1**O, 4K–L, Table 4)

Calcified tgpl borders pleomere laterally; st and stw strongly calcified (Figure 4K–L); stw merge into tgpl laterally (Figure 4K–L); ls cylindrical tapering proximally; ls carries annulated rami; ls drawn out proximo-medially and proximo-laterally into knobs forming pivot joints; ls not fully calcified, only median and lateral sides calcified and are, hence, deeply purple in the Alizarin-Red stained specimens (Figure 4K–L).

Two bulging sclerites are present in the articulation membrane of pleopods 1–5 (Figure 4K–L; Table 1). Sclerite 1 lies latero-posteriorly in the articulation membrane and is semicircular. Its anterior side forms the pivot joint with the tgpl (Figure 4K; white arrowhead; Table 1). The postero-distal part of sclerite 1 forms a pivot joint with the ls (Figure 4I, white arrowhead; Table 1). Sclerite 2 lies medio-posteriorly and is semicircular (Figure 4K–L). Postero-medially, it is drawn out proximally forming a pivot joint with st (Table 1). Distally, sclerite 2 forms a pivot joint with the ls (Table 1). The two sclerites lie next to each other on the posterior side of the articulation membrane. No apodemes were present (Table 1). Although the first pleopods form a petasma in males (as is autapomorphic for Dendrobranchiata) no differences concerning the studied structures were found in the two sexes.

### Euphausiacea

#### *Euphausia superba* Dana, 1850 (Figures **1**E, 4M–N; Table 4)

Calcified tgpl borders pleomere laterally; st and stw less calcified than tgpl (Figure 4M–N); stw merge into tgpl laterally (Figure 4F–G); ls truncated cone, with slender proximal part carrying multi-annulated rami; proximal margin of ls relatively straight on anterior side (Figure 4M), whereas uneven posteriorly (Figure 4N); ls drawn out proximo-medially and proximo-laterally into knobs forming pivot joints (Figure 4N; black arrowhead).

Three sclerites lie in the articulation membrane of pleopods 1–5 of *Eu. superba* (Figure 4M–N; Table 1). Sclerite 1 lies laterally (Figure 4M–N). Its anterior part has a straight proximal margin, whereas the distal margin is zigzag, the most distal one of the three cusps articulates with the ls via a pivot joint (Figure 4M, black arrowhead; Table 1). Sclerite 1 is drawn out postero-laterally and forms a pivot joint with the tgpl (Figure 4N; white arrowhead; Table 1). The distal margin on the posterior side is also somewhat zigzag, giving space for the lateral curve of the limb stem (Figure 4N). Sclerite 2 lies medially (Figure 4M–N). Antero-medially, it articulates with the st via a pivot joint (Figure 4M; white arrowhead; Table 1). The pivot joint of sclerite 2 and the ls is sited directly distal to this articulation (Figure 4M, black arrowhead). Posteriorly, sclerite 2 is gently excavated proximally to give space for the median parts of sclerite 1 as well as distally giving space for the tip of the ls and forming a pivot joint there with it (Figure 4N; black arrowhead; Table 1). Sclerite 3 lies on the posterior side between sclerite 1 and sclerite 2 proximal to the ls (Figure 4N). It starts with a relatively broad lateral side, extends further medio-distally, and ends in a tipped median side (Figure 4N). No apodemes were present (Table 1). Although pleopods 1 and 2 form a petasma in males, no differences in sclerite morphology were present in the two sexes.

#### *Thysanopoda tricuspidata* Milne-Edwards, 1837 (Figures 1P, 4O–P; Table 4)

Calcified tgpl borders pleomere laterally; st and stw compact and calcified (Figure 4F–G); stw merge into tgpl laterally (Figure 4F–G); ls truncated cone carrying multi-annulated rami. The proximal margin is relatively straight on the anterior side (Figure 4O), whereas it is uneven posteriorly (Figure 4P). Proximo-medially and proximo-laterally the ls is drawn out into knobs forming pivot joints.

Three sclerites are present in the articulation membrane of pleopods 1–5 of *T. tricuspidata* (Figure 4O–P; Table 1). Sclerite 1 lies laterally, is semicircular, and drawn out proximally where it forms a joint with the tgpl (Figure 4O–P; white arrowhead; Table 1). Distally, it forms a pivot joint with the ls (Table 1). Sclerite 2 lies medially (Figure 4M, N). It articulates with the st antero-medially (Table 1). Posteriorly, sclerite 2 is gently excavated distally giving space for the tip of the limb stem and forming a pivot joint with it there (Table 1). Sclerite 3 lies on the posterior side between sclerite 1 and sclerite 2 proximal to the limb stem (Figure 4N). From posterior it appears like a curved spindle (Figure 4P). No apodemes are present (Table 1). Only females were available, thus no statement for males of *T. tricuspidata* can be made.

### Lophogastrida (Figure **5**A–D)

#### *Gnathophausia gigas* Willemoes-Suhm, 1873 (Figures 1F, 5A, B; Table 4)

Calcified tgpl borders pleomere laterally; st and stw strongly calcified (Figure 5A–B); stw merge into tgpl laterally (Figure 5A–B); ls bulgy cylinder carrying multi-annulated rami; proximal end of ls drawn out laterally ending in long outgrowth nested deeply in articulation membrane (Figure 5A–B).

Five sclerites lie in the articulation membranes of the pleopods (Figure 5A–B; Table 1). Sclerite 1 is sited laterally (Figure 5A–B). Its anterior part is tipped, points anteriorly, and articulates with the tgpl (Figure 5A; white arrowhead; Table 1). Laterally, sclerite 1 arches distally lying right between tgpl and ls (Figure 5B). From there it extends further medially on the posterior side as a roundly W-shaped structure forming a pivot joint with the ls (Figure 5B; black arrowhead; Table 1). Sclerite 2 lies medially and encompasses the ls there forming a median pivot joint with it (Table 1). From anterior and from posterior it is triangular, forming altogether a rounded rhombus (Figure 5A–B). Medio-posteriorly, sclerite 2 forms a pivot joint with the st (Table 1). In the antero-posterior middle a proximo-distally oriented row of densely set setae is located (Figure 5A–B; white star). Sclerite 3 lies anteriorly in the articulation membrane and is sub-rectangular (Figure 5A). Sclerite 4 lies posteriorly between sclerite 1 and sclerite 2 (Figure 5B). Its complex shape is caused by a distal loop, visible in Figure 5B. Proximal to this loop lies a latero-medially orientated bar, which is tipped on both ends. From this bar arises another proximo-distally orientated bar, which is nested deeply in the articulation membrane. Sclerite 5 lies latero-posteriorly and is hidden by sclerite 1. It is oval (Table 1). Apodemes were found at sclerite 3, 4, and 5 (Table 1). No differences were found in the two sexes.

#### *Lophogaster typicus* Sars, 1857 (Figures 1I, 5C–D; Table 4)

Calcified tgpl borders pleomere laterally; st and stw strongly calcified (Figure 5C–D); stw merge into tgpl laterally (Figure 5C–D); ls truncated cone carrying multi-annulated rami; ls laterally and medially grown out forming pivot joints (Figure 5D; black arrowheads; Table 1), lateral outgrowth being the much larger one.

Five sclerites lie in the articulation membrane of the pleopods (Figure 5C–D; Table 1). Sclerite 1 lies laterally and is tripartite: it has a tipped anterior part, which forms a pivot joint with the tgpl (Figure 5C; white arrowhead; Table 1). The bulgy middle part lies most laterally and contacts the tgpl at the lateral extension (Figure 5D) and the ls at its lateral side. Both sclerite 1 and ls being articulated to each other there (Figure 5D; black arrowhead; Table 1). The posterior part is sub-oval and extends medially (Figure 5D). Sclerite 2 lies medially and consists of a strongly calcified distal part exhibiting a tip that points medially and forms a pivot joint with the limb stem (Figure 5C–D; black arrowheads; Table 1). The proximal part is less calcified, identifiable by its brighter purple colour, and runs towards the st forming a pivot joint with it (Figure 5D; Table 1). Sclerite 2 carries few setae pointing medially (Figure 5C–D; white stars). Sclerite 3 lies anteriorly in the articulation membrane and looks like a filled eight from anterior (Figure 5C). Sclerite 4 lies posteriorly between sclerite 1 and sclerite 2 (Figure 5D). It is L-shaped, its lower side being latero-medially orientated and lying between sclerite 1 and the st (Figure 5D). The upper part is proximo-distally orientated and contacts the ls (Figure 5D). The oval sclerite 5 lies latero-posteriorly (Table 1) and is overlapped by sclerite 1. Sclerite 3 has a large apodeme (Table 1). Another apodeme is found at sclerite 4 and 5 (Table 1). No differences were present in the two sexes.

### Mysida (Figure **5**E–F)

#### *Mysis* sp. (Figures **1**J, 5E–F; Table 4)

Females of Mysida have rudimentary pleopods consisting only of one portion and being comparably small. The same applies to pleopods 1, 2, and 5 of male mysids. Pleopods 3 and 4, however, are modified in males into a petasma for sperm transfer and are comparably large. According to these two various states the morphology of the sternites and sclerites differs. First, we describe the morphology occurring in female *Mysis* sp. and pleomeres 1, 2 and 5 in males. Then we describe the morphology appearing in the petasma of male *Mysis* sp.

*Female 1–5; male 1, 2, 5. –* The tgpl emerges from the ventro-lateral side of the segment. It extends further posteriorly, still running ventrally. In the posterior quarter of its length, the tgpl bends concavely laterally. Centrally, the less calcified sternite is sited, which is drawn out into posterior sternitic wings. The pleopod consists only of one portion and is club-shaped with a rounded tip. The proximal margin is laterally excavated on the anterior side, but rather straight posteriorly and drawn out medially. The ls is drawn out into knobs forming pivot joints proximo-medially and proximo-laterally.

Two sclerites are present in the articulation membrane of the pleopods of females and males (only pleopods 1, 2, and 5) (Table 3). Sclerite 1 lies laterally and is drop-shaped in ventral view, the tip pointing laterally. It forms a pivot joint with the limb stem antero-laterally (Table 1). Sclerite 2 lies medially and is sub-rectangular being latero-medially orientated. It forms a pivot joint with the limb stem antero-medially (Table 1). No apodemes were present (Table 1).

*Male 3–4. –* The tgpl emerges from the ventro-lateral side of the segment. It extends further posteriorly, still running ventrally. In the posterior quarter of its length, the tgpl bends concavely laterally (Figure 5E). The st is rhombic, one tip pointing anteriorly. The anterior margins are concave and end in the two lateral tips (Figure 5E). From there, the more concavely bent posterior margins run medio-posteriorly, forming a kind of bar-like posterior end (Figure 5E). From there, semicircular posterior wings arise running laterally towards the tgpl (Figure 5F). Pleopod 3 is rather large compared to one-portioned other pleopods and its limb stem is cylindrical and carries one ramus. Proximo-medially and proximo-laterally the ls is drawn out into knobs forming pivot joints. Pleopod 4 is approximately 6 times as long as pleopod 3 and has also a cylindrical ls, which carries the endopod and a long exopod, eight times as long as the endopod and reaching till the uropods. Proximo-medially and proximo-laterally the ls is drawn out into knobs forming pivot joints.

Two sclerites are present at pleopods 3 and 4 in male *Mysis* sp. (Figure 5E–F; Table 1). Sclerite 1 lies laterally and is semicircular (Figure 5E–F). Antero-laterally, it is drawn out and articulates to the tgpl via a pivot joint (Table 2). Laterally, it forms a pivot joint with the ls (Table 1). Sclerite 2 lies medially and is semicircular in ventral view (Figure 5E–F). Its anterior part is, however, very deep proximo-distally (Figure 5E) whereas the posterior part is comparably slender (Figure 5F). Medio-proximally, it is drawn out and forms a pivot joint with the posterior stw (Table 2). Medio-distally, it forms an articulation with the ls (Table 1). No apodemes are present (Table 1).

### Phyllocarida (Figure **5**G–L)

The pleon of Phyllocarida is tagmatised: the anterior four segments form a unit characterised by comparably large pleopods with limb stem, endopod, and exopod and being used for swimming. In segments 5 and 6 the pleopods consist of only two portions and are much smaller than pleopods 1–4. Sclerites are only present at the bases of pleopods 1–4. Segment 7 is limbless. The last body part is the conical telson carrying large furcal rami.

#### *Nebalia bipes* (Fabricius, 1780) (Figures 1K, 5G–H; Table 4)

Calcified tgpl borders pleomere laterally; st and stw strongly calcified (Figure 5G–H); posterior stw set-off from st (Figure 5H); ls cylindrical carrying spine-bearing rami; proximal anterior margin of ls convex (Figure 5G); whereas posterior margin rather concave (Figure 5H); ls drawn out proximo-medially and proximo-laterally into knobs forming pivots.

Eight sclerites are present in the articulation membranes of pleopods 1–4 (Figure 5G–H; Table 1). Sclerite 1 lies antero-laterally (Figure 5G–H). It is elongated and its main part extends anteriorly (Figure 5G). Sclerite 1 encompasses the ls laterally, by what a small part of sclerite 1 lies also posteriorly (Figure 5H). Sclerite 1 forms an articulation with the tgpl and the ls (Table 1). Sclerite 2 lies medially, is C-shaped and curves around the ls (Figure 5G–H). The whole distal margin of sclerite 2 runs over the ridge of the st (Figure 5H). Proximally, it forms an articulation with the ls (Table 1). The sub-rectangular sclerite 3 lies laterally in the articulation membrane between sclerite 1 and the limb stem (Figure 5G). Sclerite 4 lies laterally to sclerite 2 on the posterior side of the articulation membrane (Figure 5H). It is sub-oval with a latero-distal outgrowth (Figure 5H); sclerite 4 is tripartite in pleopod 1. Sclerite 5 is sited lateral to sclerite 4 and right proximal to the margin of the ls (Figure 5H). It is crescent-shaped. The sub-rectangular sclerite 6 is sited distal to sclerite 5 and is flanked latero-posteriorly by sclerite 7 and medially by the elongated sclerite 8 (Figure 5H). The single sclerites, however, stick very closely together and fit like puzzle pieces into each other. Apodemes are present at sclerite 3 and sclerite 4 (Table 1). No differences were found between the two sexes.

#### *Nebaliopsis* sp. (Figure 1L, 5I–L; Table 1)

Calcified tgpl borders pleomere laterally; st lies medially lies (Figure 5I–J); stw set off from st (Figure 5I–J); ls cylindrical carrying rami; ls drawn out proximo-medially and proximo-laterally into knobs forming pivots (Figure 5J; white arrowheads).

Six sclerites are present in pleopods 1–3 (Figure 5I–J; Table 1). Sclerite 1 lies laterally and extends further medially into the anterior articulation membrane (Figure 5I). It is C-shaped and encompasses the ls laterally (Figure 5J). It articulates to the tgpl (Figure 5J, white arrowhead; Table 1) and the ls (Figure 5J, black arrowheads; Table 1). Sclerite 1 is centrally located, sub-rectangular and latero-medially oriented. Laterally it contacts sclerite 2. Sclerite 2 lies medially (Figure 5I–J). It is C-shaped and encompasses the ls, and articulates to st and ls (Figure 5J; white arrowhead; Table 1). Sclerite 2 is C-shaped, lies laterally and articulates with tgpl and ls. On the posterior side a single large, in ventral view drop-shaped sclerite 3 is present (Figure 5L). Sclerite 3 lies laterally to sclerite 2, but still in the median half of the posterior articulation membrane (Figure 5J). It is sub-triangular and medially overlapped by sclerite 2 (Figure 5J). Sclerite 4 lies latero-distally and is sub-rectangular with rounded edges (Figure 5J). Sclerite 5 is comparably large and lies centrally in the posterior part of the articulation membrane (Figure 5J). It consists of an elongated, crescent-shaped median part and a semicircular lateral part being attached to it (Figure 5J). Proximal to sclerite 5 is sclerite 6, which is triangular and contacts sclerite 5 and the tgpl (Figure 5J). Only three sclerites are present in the articulation zone of pleopod 4 (Figure 5K–L): Anteriorly, two sclerites lie in the articulation membrane. Sclerite 1 is centrally located, sub-rectangular and latero-medially oriented. Laterally it contacts sclerite 2. Sclerite 2 is C-shaped, lies laterally and articulates with tergopleura and limb stem. On the posterior side a single large, in ventral view drop-shaped sclerite 3 is present (Figure 5L).

The presence of apodemes was not examinable. No differences between the two sexes were noted.

### Stomatopoda/Hoplocarida (Figure **5**M–P)

#### *Erugosquilla massavensis* (Kossmann, 1880) (Figure 1D, Figure 5M–N; Table 4)

Calcified tgpl borders pleomere laterally; st and stw strongly calcified (Figure 5M–N); stw merge into tgpl laterally; ls short, slightly compressed cylinder, ls carries multi-annulated ex also a gill and the equally multi-annulated en; anterior and posterior margins of ls slightly concave (Figure 5M, N); ls drawn out proximo-medially and proximo-laterally into pivots.

Eight sclerites are present in the articulation membranes of the pleopods (Figure 5M–N; Table 1). Sclerite 1 lies antero-laterally (Figure 5M–N). It is elongated and the anterior part is somehow dolphin-shaped in ventral view (Figure 5M). However, laterally it extends into a C-shaped part, which encompasses the limb stem and extends onto the posterior side of the articulation membrane (Figure 5M–N). This is also the part forming articulations with the tgpl (Figure 5N; white arrowhead; Table 1) and the ls. Sclerite 2 lies medially and is C-shaped. It is built of a small anterior part (Figure 5M) and a rather bulgy part followed by a comparably slender part extending further laterally until the extension of the st on the posterior side (Figure 5N). The sternitic extension and the postero-lateral end of sclerite 2 form an articulation (Table 1). It is not connected to the median knob of the pleopod insertion (Figure 5M). Sclerite 3 and sclerite 4 are comparably small, oval, and lie distal to sclerite 1 at the anterior side (Figure 5M). A fifth, equally small sclerite 5 lies further laterally being hidden in a membrane fold (Figure 5M; black arrow). Posteriorly, an elongated sclerite 6 contacts sclerite 1 with its lateral end and sclerite 2 medially (Figure 5N). Proximal to sclerite 6 lies the sub-rectangular sclerite 7, which is proximally surrounded by the L-shaped sclerite 8 (Figure 5N). Apodemes were present on the insides of sclerites 1, 4, 6, and 8 (Table 1). No differences were found between the two sexes.

#### *Gonodactylus chiragra* (Fabricius, 1781) (Figure 1G, 5O–P; Table 4)

Calcified tgpl borders pleomere laterally; tgpl starts laterally on anterior side of the pleomere adjoining former segment and extending latero-posteriorly where curving dorsally forming a latero-posterior tip; st and stw strongly calcified (Figure 5O–P); stw merge into tgpl laterally; ls short, slightly compressed cylinder carrying annulated ex, a gill and equally multi-annulated en; anterior and posterior margin of ls concave (Figure 5O–P); ; ls drawn out proximo-medially and proximo-laterally into knobs forming pivots.

Eight sclerites are present in the articulation membranes of the pleopods (Figure 5O–P; Table 1). Sclerite 1 lies antero-laterally (Figure 5O–P). It is elongated and its main part lies anteriorly (Figure 5O). Sclerite 1 encompasses the limb stem laterally, by what a small part of sclerite 1 lies also posteriorly (Figure 5P). Sclerite 1 forms an articulation with the tgpl and ls (Figure 5P; white arrowhead; Table 1). Sclerite 2 lies medially and is C-shaped encompassing the ls (Figure 5O–P). Its anterior part is very long and is located very close and proximal to the limb stem. Sclerite 2 articulates with the ls but it does not contact the median knob in the insertion of the pleopod (Figure 5O–P; Table 1). Sclerite 3 and sclerite 4 are comparably small, oval, and lie distal to sclerite 1 at the anterior side (Figure 5O). A fifth sclerite 5 is present antero-medially being elongated and running parallel to the margin of the pleopod insertion (Figure 5O). Posteriorly, the elongated sclerite 6 contacts sclerite 1 with its lateral end and sclerite 2 medially (Figure 5P). Proximal to sclerite 6 lies the sub-rectangular sclerite 7 (Figure 5P). Proximal to sclerite 7 lies the sub-oval sclerite 8. Apodemes were found on the insides of sclerites 1, 4, and 6 (Table 1). No differences are present between the two sexes.

### Reconstruction of the ground-pattern states for the sclerite conditions of taxa within Malacostraca

Calcified sclerites occur in the pleopod-body arthrodial membrane in all examined species (Figures 2–5; Table 1). They occur in constant number and position within one species on pleopods 1–5 (resp. on pleopods 1–4 in phyllocarids). As an exception, in species with a tagmatised pleon the sclerite pattern is at least similar in one tagma, e.g. *Dikerogammarus haemobaphes* and *Hyperia* sp. (Figure 4A–H for Amphipoda; or *Nebalia bipes* and *Nebaliopsis* sp. Figure 5G–L for Phyllocarida). The sclerite patterns are even similar in representatives of one supra-specific taxon (Table 1; Table 4). Therefore, the sclerites seem to be very conservatively preserved structures, as they appear even in strongly modified pleopods, for instance gonopods forming petasms in, e.g., *Paranaspides lacustris* (pleopods 1–2; Figure 3C) or *Mysis* sp. (pleopods 3–4; Figure 5E–F). Almost no literature includes detailed information on this character complex for comparisons. Exceptions are rare [29]. These species-specific data (Table 2 and Table 3) formed the basis for a reconstruction of the ground-pattern states with regard to the sclerites of all major malacostracan taxa.

*Sclerites in the ground pattern of Anaspidacea*. – In the three species of Anaspidacea – *Anaspides tasmaniae*, *Allanaspides hickmani*, *Paranaspides lacustris –* there is one lateral sclerite, that is anteriorly bifurcate and forms pivot joints with both the tergopleura and the basipod (sc 1 in Figures 2, 3; Table 1). Another joint is formed by basipod and sternite (Figures 2, 3; Table 1). Despite the different life styles of these species [4,30,31] we find great similarities in limb morphology. We conclude that a single, lateral, anteriorly bifurcate sclerite forming a pivot joint with the tergopleura and extending comparably much into the posterior articulation membrane was present in a common stem species and is therefore part of the ground pattern of Anaspidacea (Table 2; Table 3).

*Sclerites in the ground pattern of Amphipoda. –* The lateral sclerites 1 are semicircular and form pivot joints with tergopleurae and basipods in both species (Figure 4B, F, black and white arrowheads; Table 1). They do not extend much into the posterior articulation membrane. The median sclerites 2 are semicircular and form joints with sternites and basipods in both species (Figure 4B–C, F–G; Table 1). Interestingly, the median sclerites of both species have a proximal outgrowth forming the joint with the sternite (Figure 4B, F, G, white arrowhead, right ones respectively; Table 1). The anterior sclerites 3 differ in shape and position in the two species (Figure 4B, F). Also the distal, posterior sclerites 4 vary enormously in shape (sc 4 in Figure 4C, G), but the more proximal sclerites 5 again are very similar in shape and position (Figure 4C, G). The proximal, posterior sclerite 5 has an apodeme in *D. haemobaphes* (Table 1).

Amphipoda is the only taxon included in this study, of which the pleopod-body articulation has been examined in detail. [29] examined *inter alia* "sclerotised plates" (p. 584) of *Eurythenes gryllus* (Lichtenstein in Mandt, 1822). Despite the accurate documentation there, lack of labelling complicates homologisation with our results. Yet, *Eur. gryllus* seems to have 5 sclerites as well ([29], Figures 3 and 4) and two of its sclerites correspond to our anterior sclerite 3 and posterior sclerite 5 both with apodemes (see his figure 4B insertion of M6 = apodeme sclerite 3; figure 4A insertion of M8 = apodeme sclerite 5).

We conclude that the ground-pattern for Amphipoda includes five sclerites (Table 2 and Table 3): a large lateral one forming joints with the tergopleura and the basipod, a large median one also forming joints and having a proximal outgrowth, an anterior third sclerite between lateral and median sclerite, and two posterior sclerites lying compactly aggregated rather laterally. Based on the data of [29] and our observations on *D. haemobaphes* we assume there is an apodeme for the anterior sclerite and the proximal sclerite on the posterior side (Table 1, Table 2 and Table 3). We reconstruct this ground-pattern state despite the various life styles realised in different amphipod species (e.g. [23,29,33]).

*Sclerites in the ground pattern of Decapoda*. – In Decapoda (sensu lato, including *Amphionides reynaudii* H. Milne Edwards, 1832 [34]), *Neosergestes semissis* has three, *Penaeus monodon* two sclerites (Figure 4J–L; Table 1), thus there are two states for the sclerite pattern complicating a ground pattern reconstruction for this taxon. Yet, the lateral and median sclerites are very similar in both species: The lateral sclerites 1 are semicircular and form joints with tergopleurae and basipods and exhibit an apodeme (Table 1). The median sclerites 2 are both semicircular and form joints with sternites and basipods; the sternite-sclerite 1 joint is shifted posteriorly (Table 1). In both species, the lateral and median sclerites touch each other posteriorly (Figure 4J, L), which means they extend comparably far into the posterior articulation membrane. Remarkable is also that the lateral sclerite in decapods does not extend much into the anterior articulation membrane compared to all other species that we examined (except for lophogastrids). The presence of a third posterior sclerite in the stem species of Decapoda as visible in *Neo. semissis* seems likely. Yet, this can only be revealed by an out-group comparison. Depending on the phylogenetic position assumed for Decapoda, two groups are favoured for an out-group comparison: either Euphausiacea (suggested as sister taxon in [35]), or Xenommacarida (proposed in [36]). Both of these presumptive sister taxa have a posterior sclerite (see below), which is why we assume it also is true for Decapoda (Table 2).

*Sclerites in the ground pattern of Euphausiacea*. – The sclerite situation in both *Euphausia superba* and *Thysanopoda tricuspidata* are very similar: the lateral sclerites 1 are very similar in shape (note the bulginess and especially the anterior zigzag, Figure 4M, O) and form pivot joints with the tergopleura (Figure 4N–P, white arrowheads) and basipods. The median sclerites 2 are equally similar (Figure 4N, P) and form joints as well (Figure 4N, black arrowhead). In both species sclerites 1 and 2 fit perfectly into each other posteriorly (Figure 4N, P), which means they extend comparably far into the posterior articulation membrane. Distal to this contact area, a third sclerite is present. The similarity in the sclerite pattern is probably as well due to the similar life styles of both examined species [23,24]. We assume a ground pattern state comprising three bulging sclerites without apodemes for Euphausiacea (Table 2 and Table 3).

*Sclerites in the ground pattern of Lophogastrida. –* In both *Gnathophausia gigas* and *Lophogaster typicus,* we found five sclerites: Large lateral ones forming anteriorly shifted pivot joints with the tergopleura laterally (sc 1 in Figure 5A, C, white arrowheads for joints; Table 1) and, via a medially pointing outgrowth, the basipod medially (Figure 5B, D, black arrowheads). Median sclerites 2 form posteriorly shifted pivot joints with the sternites (Table 1), with the basipods, and exhibit setae (Figure 5A–D, white stars). Both species have a small anterior sclerite 3 (Figure 5A, C) and a complexly shaped sclerite 4 posteriorly (sc 4 in Figure 5B, D), both having internal apodemes (Table 1). Sclerite 5 lies latero-posteriorly and has another apodeme (Table 1, Table 2 and Table 3). Due to the similar life habits of lophogastrid species [23], we assume the ground pattern state for Lophogastrida correlates to the sclerite pattern description above (Table 2 and Table 3). We propose that the lateral sclerite does not extend very far into the anterior articulation membrane in comparison to the situation seen in all the other species we examined.

*Sclerites in the ground pattern of Mysida. –* Two sclerites appear on all pleopods of the examined *Mysis* sp., independent from the gender. However, the sclerites at the modified pleopods 3 and 4 for males (Figure 5E–F) are much larger than the sclerites found on other, rather reduced pleopods. We consider this significant for our reconstruction of the ground pattern (Table 2): i.e. two sclerites present in Mysida, the lateral one forming an anteriorly shifted articulation with the tergopleura; the median one has an outgrowth which forms a postero-laterally shifted articulation with the sternite; both sclerites lack apodemes. In general, all species of Mysida seem to share a similar life style [24], for which reason no drastic modifications from the ground pattern state are expected for other species.

*Sclerites in the ground pattern of Stomatopoda/Hoplocarida. –* In Hoplocarida the sclerite patterns in both examined species are very similar, for which reason the ground pattern is assumed as follows (Table 2 and Table 3): a large lateral and a large median sclerite participating in pivot joints (sc 1, sc 2 in Figure 5M, O). The lateral sclerite has an apodeme and does not extend much into the posterior articulation membrane (Figure 5N, P). Anteriorly, two small sclerites lie distally to the lateral sclerite, the more median one having an apodeme, and a slightly larger sclerite lies medially (sc 3–5 in Figure 5M, O). Posteriorly a large distal sclerite connects the median and lateral ones and has an apodeme (sc 6 in Figure 5M, O). Two smaller sclerites (sc 7 and 8 in Figure 5M, O) are sited proximal to sclerite 6. Interestingly, the Recent benthic Hoplocarida are the only Malacostraca that have gills on their pleopods [23,37]. This could explain why they have the highest number of apodemes; these might guarantee extra versatility to the movement of the pleopods and facilitate sufficient oxygenation.

*Ground pattern of Phyllocarida. –* The pleons of *Nebalia bipes* and *Nebaliopsis* sp. are tagmatised. The posterior pleopods (5 and 6) are small and appear reduced, and the sclerite pattern of pleopod 4 differs also significantly from that of the anterior three. Our reconstruction is restricted to pleopods 1–3.

The number of sclerites differs in both examined species (Figure 4G–L; Table 1 and Table 3). Nevertheless, similarities can be found, which consequently can be assumed for the common stem species (Table 2; Table 3): a large lateral sclerite and a median sclerite both forming pivot joints with sternite, tergopleura, and the basipod (Figure 4G–J, white arrowheads; Table 1; Table 3). The lateral sclerite does not extend much into the posterior articulation membrane (Figure 5H, J). An antero-lateral sclerite is only found in *Neb. bipes* (Figure 5G; Table 1; Table 3). Yet, we assume this condition is of some significance for the phyllocaridan ground pattern because similar, antero-lateral sclerites are also found in a member of the next closest relatives Eumalacostraca, the Hoplocarida (see below). For the posterior sclerites we deduce a state for the stem species in form of a large lateral sclerite (sc 6 in *Neb. bipes* Figure 4H; sc 5 in *Nebaliopsis* sp. Figure 5J; Table 3) surrounded by a smaller distal sclerite (sc 5 in *Neb. bipes* Figure 4H; sc 4 in *Nebaliopsis* sp. Figure 5J; Table 3), a smaller proximal sclerite (sc 7 in *Neb. bipes* Figure 4H; sc 6 in *Nebaliopsis* sp. Figure 5J), and a smaller median sclerite (sc 4 in *Neb. bipes* Figure 4H; sc 3 in *Nebaliopsis* sp. Figure 5J). Considering the dissection results of *Neb. bipes* (Table 1), we assume apodemes for the anterior sclerite and the medio-posterior one (Table 2). *Neb. bipes* has an additional posterior sclerite (Figure 5H; Table 1). *Nebaliopsis* sp. lacks the small anterior sclerite (Figure 5I) of *Neb. bipes*. Both conditions might be autapomorphies of the respective taxon (if they are monophyletic [38]) what should be illuminated by further studies). Differences of the examined species from the state in the ground pattern of the Phyllocarida might be explained by the different life styles: *Nebaliopsis* is a bathypelagic swimmer [39] whereas *Neb. bipes* is benthic [40].

### Phylogenetic mapping

We mapped the reconstructed ground-pattern states onto two recently published phylogenetic hypotheses [35,36] (Figure 6). Our expectation was that this should help us to reconstruct the ground-patterns of higher taxa and, with this, unravel more aspects of the evolution of the sclerite complex within Malacostraca.

Both phylogenetic hypotheses are in agreement concerning the relationship of the basal divergence of Phyllocarida and Eumalacostraca, the latter including Hoplocarida and Caridoida (Figure 6A). The position of Bathynellacea remains uncertain because in these animals the pleopods are strongly reduced, in some species even being absent (except for the uropods, see e.g. [28]). Accordingly bathynellids were left unconsidered in our study.

Taking the basal relationships of Malacostraca for granted, the ground-pattern of sclerites in both Phyllocarida and Hoplocarida is crucial for the reconstruction of the sclerite pattern in the ground pattern of Malacostraca and Eumalacostraca. In fact, the situations in Phyllocarida and Hoplocarida (Table 2: and Table 3) may be taken as basis for the reconstruction of the sclerite condition in the stem species of Malacostraca (Figure 6A; Table 3). Accordingly, our homologisation of the sclerites in relation to the malacostracan ground pattern below is given in Table 3.

*Sclerites status in the ground pattern of Malacostraca* (Figure 6A; Table 3). – For malacostracan pleopods we can assume that one comparably large sclerite lies laterally and articulates with the tergopleura and the basipod. It extends more into the anterior aspect of the arthrodial or articulation membrane than into the posterior aspect. Another large sclerite lies medially and participates in joints with sternite and basipod. At least one additional smaller sclerite lies anteriorly, located slightly lateral and distal to the lateral sclerite. In the posterior articulation membrane are four sclerites: one sclerite relatively larger than the other three, by which it is surrounded. The phyllocarids largely reflect this condition.

In Hoplocarida, the condition has slightly changed compared to that of the ground-pattern state of Malacostraca (Figure 6A; Table 3): Two additional smaller sclerites are located in the anterior joint membrane and only three sclerites (instead of four) are present posteriorly. Whether the fourth posterior sclerite became lost or fused with another cannot be said. Furthermore, the most distal of the posterior sclerites is elongated and the articulation of sternite and median sclerite has shifted anteriorly. All these features are probably autapomorphies of Hoplocarida except for the presence of only three posterior sclerites (Table 2; Table 3). The small number of posterior sclerites probably had evolved in the stem species of Eumalacostraca, as no other in-group taxon exhibits more than two posterior sclerites (Figure 6A; Table 3).

More complex and challenging is the situation within Caridoida. Tabacaru & Danielopol [35] assume a sister group relationship of Decapoda and Euphausiacea, uniting these two taxa in Eucarida (Figure 6B). The sister taxon to Eucarida (N. N. in Figure 6B) should comprise the Anaspidacea and Neocarida (Thermosbaenacea + Peracarida). Wirkner & Richter [36] on the other hand suggested Decapoda as the adelphotaxon to a taxon, which they called Xenommacarida, which comprises the Anaspidacea + N. N. (Euphausiacea + Neocarida) (Figure 6C).

*Sclerite condition in the ground pattern of Eucarida. –* The assumption of a common origin of Euphausiacea and Decapoda (Figure 6B) would imply that three sclerites were probably present in the common stem species. This suggests the presence of only one posterior sclerite in the stem species of Eucarida. As in both, Decapoda and Euphausiacea, the lateral and median sclerites extend far into the posterior articulation membrane and touch each other posteriorly, this was most likely also a feature of the eucaridan stem species because this condition is found in no other taxon and is most likely an autapomorphy of Eucarida. Yet it is unclear whether the posterior sclerite in the eucaridan stem species lies more distally as in Euphausiacea or more proximally as in Decapoda.

It is reasonable to assume that the shape of the sclerites in Euphausiacea is an autapomorphy (indicated by the red-grey marbling of the euphausiacean sch. in Figure 6B), while the anterior shortening of the lateral sclerite and the posterior shift of the median sclerite-sternite articulation are autapomorphies for Decapoda (emphasised by red colour in Figure 6B).

*Sclerite condition within Neocarida. –* Inside Neocarida relationships are heavily disputed (see e.g. [36,41,42]), so we focused on the phylogeny of Tabacaru & Danielopol [35]. In the ground patterns of all examined neocaridan taxa, Amphipoda, Lophogastrida, and Mysida, a lateral and a median sclerite is present. In all these ground patterns, the articulation between the median sclerite and the sternite is in a more posterior position than in other examined taxa. However, in the three neocaridan taxa this condition is achieved either by a posteriorly positioned simple pivot in Lophogastrida, or by a posteriorly extending outgrowth in Amphipoda and Mysida.

*Sclerites in the ground pattern of "Mysidacea"* (Figure 6D)*. –* The lateral sclerite articulates with the basipod and with the tergopleura; the latter articulation is shifted anteriorly. The median sclerite articulates with the basipod and the sternite, the latter articulation being shifted posteriorly and formed by an outgrowth of the median sclerite, as present in Amphipoda. The anterior sclerite lies slightly laterally and has an apodeme. Two posterior sclerites occur, the distal one with an apodeme.

Lophogastrida would have changed some details of this ground pattern situation (red elements in their sch. in Figure 6D): The lateral sclerite runs anteriorly and is parallel to the tergopleura; it does not bend around the basipod (Figure 6D, dashed red lines). The median sclerite bears setae and articulates directly with the sternite; not via an outgrowth. Mysida have retained only the lateral and the median sclerites. In general, Amphipoda and Lophogastrida are more similar to each other than to Mysida, both exhibiting five sclerites against two in Mysida. Yet, a closer relationship of Lophogastrida and Mysida (= Mysidacea) has been assumed [35,43] (Figure 6D) but also rejected [44-46]. A further reconstruction of sclerite patterns for Neocarida is uncertain, hindered by the absence of data from and taxa within Mancoida, in part caused by lack of pleopods (most likely reduction) in many in-groups, e.g. Cumacea, and unclear in-group relationships [36]. Adequate out-group comparison is not possible, because Thermosbaenacea have only reduced pleopods 1 and 2 and these appear to lack any sclerites (personal observations). The state most parsimoniously assumed for Peracarida is also valid for Neocarida as no further character polarisation can be performed with Thermosbaenacea (Figure 6D, question mark). We conclude for the ground pattern of Neocarida and/or Peracarida that the lateral sclerite is involved with the articulations with the basipod and tergopleura; this sclerite does not extend as much into the posterior articulation membrane as it does into the anterior one. The median sclerite articulates with the basipod and sternite, the latter articulation being shifted posteriorly and formed by an outgrowth of the median sclerite. One sclerite with an apodeme lies in the anterior articulation membrane; two other sclerites lie posteriorly, the proximal one having an apodeme.

The enormous extension of the lateral and median sclerites into the anterior articulation membrane found in Amphipoda is regarded as an autapomorphy of this taxon (red elements in their sch. in Figure 6D), albeit depending on the state in Mancoida.

The reconstruction of a possible ground-pattern of the sclerites for N. N. (Anaspidacea + Neocarida, Figure 6B) is difficult because the state in Anaspidacea differs enormously from the one found in our sampling of Neocarida. Compared to the sclerite state assumed for Eucarida, the stem species of N. N. must have featured at least the following postulated character states.

*Ground-pattern for N. N. (Anaspidacea + Neocarida). –* The lateral sclerite forms a central articulation with the tergopleura and the basipod. It extends comparably far into the posterior articulation membrane. The median sclerite articulates via pivot joints with sternite and basipod, both being located centrally. One other sclerite lies posteriorly. Whether this latter sclerite had an apodeme remains uncertain, due to character-state polarisation with Hoplocarida.

This implies that the single, anteriorly bifurcate sclerite in Anaspidacea, which forms an anteriorly shifted joint with the tergopleura and the median articulation is formed by the direct contact of the sternite and basipod, are autapomorphies of Anaspidacea (Figure 6B). Consequently, autapomorphies of Neocarida would then have to be: the posterior shortening of the lateral sclerite, the addition of an anterior sclerite with apodeme, the addition of a laterally shifted posterior sclerite, the lateral shift of the remaining posterior sclerite, and the posterior shift of the median articulation of the median sclerite, and the sternite developed as an outgrowth.

The ground-pattern states concerning sclerites of Eucarida and N. N. (Anaspidacea + Neocarida) are almost identical. This is why we can assume the following sclerite state for Caridoida.

*Ground pattern of Caridoida. –* The lateral sclerite forms a central articulation with the tergopleura and the basipod. It extends relatively far into the articulation membranes, anteriorly and posteriorly. The median sclerite forms a central joint with sternite and basipod. If lateral and median sclerite are touching each other posteriorly is unclear. Posteriorly another sclerite is present, but whether it exhibits apodemes remains also uncertain.

Based on what we reconstruct for the eumalacostracan state (Figure 6A), this would imply the following autapomorphies for Caridoida: 1) the extension of the lateral sclerite into the posterior articulation membrane, 2) the loss of the anterior sclerite, 3) the loss of two posterior sclerites, and 4) the median shift of one of the posterior sclerites.

However, this reconstruction does not seem parsimonious because the conditions found in the eumalacostracan and neocaridan stem species would appear rather similar because: 1) the lateral sclerite extends more into the anterior articulation membrane than into the posterior one, 2) the presence of an anterior sclerite with apodeme, and 3) the appearance of more than one posterior sclerite in both. These features are absent in our reconstruction of the caridoid ground pattern (Figure 6B). Thus, Neocarida would have to have made a step back and re-evolved these features, which is not parsimonious.

We have a similar situation when mapping our ground-pattern reconstructions on the phylogeny of Wirkner & Richter [36] (Figure 6C): We cannot unambiguously polarise the scleritic situation in Anaspidacea vis-à-vis a common stem pattern with Euphausiacea and Neocarida (Figure 6C). We would have to assume three sclerites as the most parsimonious condition, the lateral sclerite extending far into the anterior and posterior articulation membranes. Consequently, the inflated condition of the euphausiacean sclerites would be an autapomorphy, as would be the posterior shortening of the lateral sclerite, the presence of an anterior sclerite with apodemes, and an additional posterior sclerite in Neocarida. It is most parsimonious to assume that the stem species of Xenommacarida possessed the same condition as seen in N. N. (Euphausiacea + Neocarida) (Figure 6C), as well Caridoida. This would mean that in Anaspidacea the situation autapomorphically reduced to the single and anteriorly bifurcate sclerite, and the median articulation directly with the basipod and sternite evolved autapomorphically. In Decapoda the anterior extension of the lateral sclerite and the posterior position of the median articulation would be autapomorphic. The autapomorphies of Caridoida would be the loss of the anterior sclerite, as well as of two posterior ones, the median shift of one posterior sclerite, and the posterior extension of the lateral sclerite.

## Discussion

### Value of sclerite patterns

Calcified sclerites appear to occur at the base of the pleopods, at least for the malacostracan taxa we investigated. Some of the sclerites form pivot joints between the sternites medially and tergopleurae laterally. The limb stem forms corresponding depressions or knobs proximally. In addition, some internal apodemes act as attachment sites for muscles. The pattern of the sclerites is not only rather invariable between individuals of a species and within the series of pleopods of a species, but also conservative within members of putatively monophyletic groups. Accordingly we were able to reconstruct ground-pattern states of the according taxa.

Mapping our data onto existing phylogeny hypotheses for Malacostraca mirrors the basal sister-group relationship of Phyllocarida and Eumalacostraca, long suggested before [31,35,36,41-43,47,48], but see, e.g. [49]. Phyllocarida possess the highest number of sclerites, four well-developed lateral and median joints and no dislocation of these (Table 2), which we assume represents the ancestral condition of this arrangement. This pattern of sclerites overlaps with that of Hoplocarida (Table 2), in most hypotheses turning out as the next branch in the system of Malacostraca and sister taxon to Caridoida. Accordingly we regard the sclerite pattern of Phyllocarida as the least derived one and reflecting much of the character condition in the ground pattern of Malacostraca.

Compared to this the number of sclerites and joint locations is slightly smaller but still large in the caridoid taxa Lophogastrida and Amphipoda among the Peracarida, both having five sclerites (Table 2). Within Peracarida, the presence of five sclerites in Amphipoda points to a rather basal position of this taxon. This differs from the phylogeny proposed by Wirkner & Richter [36], Tabacaru & Danielopol [37], and [50], in which Amphipoda are deeply nested within Peracarida. However, the sclerite pattern is congruent with the peracaridan system promoted by Kobusch [51], in which Lophogastrida represent the sister taxon to all other Peracarida, comprising Amphipoda and the remaining taxa Mysida and Mancoida. Following this, Mysida exhibit the most derived condition with two sclerites of the three peracaridan taxa investigated, but interestingly linked to amphipods by a shared extension of one of the sclerites to the sternite, pointing to their separation from Lophogastrida (Table 2). Also in this respect, our data fit with the phylogeny of Kobusch [51]. The scleritic extension to the sternite (Table 2) could represent a specific marker of evolutionary changes within the Peracarida, but this has to be investigated further in the remaining mancoidan in-group taxa as much as the low number of sclerites, which might rather be taxon-specific due to the high reduction of the pleopods in Mysida.

Within the remaining Caridoida, Euphausiacea, and Decapoda share a very specific sclerite pattern (Table 2). This hints at their sister-group relationship [35,48]. Here it is even more strongly supported by no less than three details in the arrangement: lateral and median sclerites touch each other; absence of the anterior sclerite; no posterior sclerite (Table 2). Dislocation of the pivot joints, either to the tergopleura, or to the sternite, could be taxon-specific and independently derived due to functional adaptations (Table 2).

The pivot joint between lateral sclerite and tergopleura occurs in the same fashion throughout all investigated taxa, hence appears to be conservatively retained from the ancestral state = ground-pattern of Malacostraca. This is true even for the Anaspidacea, in which, otherwise and autapomorphically, an antero-lateral extension of the bilobed lateral sclerite articulates with the tergopleura instead of a simple pivot joint (Table 2). Also in other details, the situation in Anaspidacea, with only a single but bifurcate sclerite, is autapomorphic, a condition most likely associated with the specific orientation and movements of the pleopods, i.e., the limbs are oriented laterally during swimming of *Anaspides tasmaniae*.

Summing up, our initial investigations of the sclerite pattern in representatives of major malacostracan taxa demonstrate the value of this character complex, and potential as a phylogenetic marker for malacostracan in-group phylogeny. In addition to the petasma [52,53] and uropod morphology [28], the sclerite pattern represents a third morphological complex associated with the pleon available for more detailed investigations and analyses in the future. This is especially important since [42] demanded for new character sources for resolving (eu)malacostracan relationships.

### Origin of the calcitic sclerites

Topologically, the sclerites on the malacostracan pleopods appear to be located within the basal arthrodial membrane, i.e., between the sclerotised ventral body proper and sclerotised limb stem. Clearly all malacostracans possess a coxal limb portion on all their anterior eight thoracopods. Phyllocarida have antero-posteriorly flattened limbs with likewise flat and large coxal and basipodal portions. The well-sclerotised and calcified coxae of all other taxa, which are generally better calcified than those in phyllocarids (speaking only of the living taxa here) are fairly short and possesses a well developed pivot joint medially and laterally. Another such pair of pivot joints connects coxa and basipod, more the endopodal articles. This articulation system includes membranes between all sclerotised portions, including the basal area. Even rather modified thoracopods share this morphology rather conservatively and in a rather uniform design. This suggests that a change from having neither a proximal endite nor a coxa should have occurred before or with the stem species of all modern taxa, therefore the evolutionary transition remains obscure.

Assuming that the calcitic sclerites on the malacostracan pleopods correspond to the phylogenetically old median soft and setae-bearing proximal endite (feature of Crustacea s. l., see [10]), this endite should have become sclerotised, calcified and split into several sclerites before the evolution of the malacostracan stem species – and this in line with the *de novo* formation of pivot joints. The start from a euarthropod limb situation lacking a proximal endite or coxa is likewise improbable since it also requires the evolution of the sclerites and pivots *de novo*.

At least it remains clear that four pivots have therefore to be expected also for pleopods if they have or had a coxal portion, and moreover two membrane areas. This scenario assumes a long series of thoracopods in the malacostracan stem species, all equipped with a coxa-basipod limb stem. With the tagmotic distinction into two sets and functional deviation of the two sets of limbs, also their morphology became more distinct from that of the anterior set. One is the appearance of calcified sclerites, as investigated here, instead of a well-visible coxa body. Yet pleopods also retained conservative traits. The most apparent is the retention of the pivots. The proximal and distal outlines of the sclerites form the minimum range of the supposed coxal area, because these are located within the sclerotised coxal cuticle.

**Figure 7:**
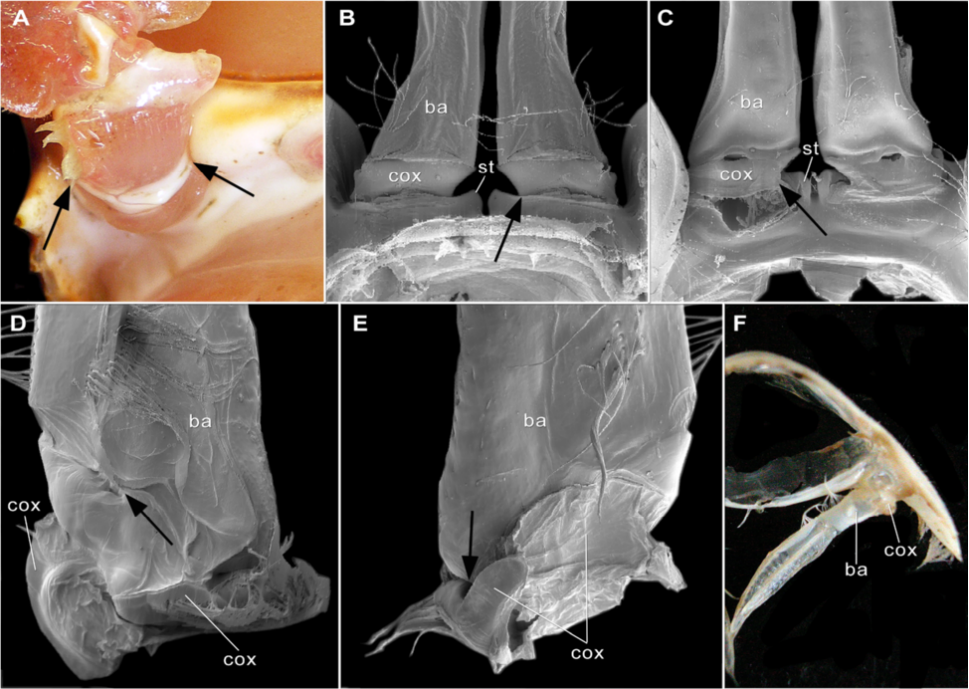
Images of the basal part of pleopods. Abbreviations as before. **A**. *Palinurus elephas* Latreille, 1803. Note that the sclerites are separated from each other by a small membranous area and almost solely reflect the outline of the coxa. **B, C**. *Gammarus roeselii* Gervais, 1835. The limb stem comprises the short coxa (artificially highlighted by shrinking effects during the drying process for SEM) and the elongated tubular basipod (cf. Figure 4A–H). **B**. Posterior view. **C**. Anterior view. Note that the coxal outline is rather well determinable. **D**, **E**. *Nebalia bipes* (cf. Figure 5G–L). **D**. Posterior view. **E**. Anterior view. The coxal outline is not as clear as in the amphipod. **F**. Pleomeric segment of the crayfish *Astacus leptodactylus* exhibiting the coxal portion of the pleopod. Note that the coxal outline is almost only reflected by the presence of sclerites.

All membrane between these lines is unsclerotised coxal cuticle, not belonging to the true joint membranes. These softer cuticular areas lie either anteriorly or posteriorly of the limb, while the basipod may have a deep excavation to enlarge the joint area only posteriorly (Figure 7A). According to the degree of softness, the coxa may be more or less effaced in this outline, which may be the main reason to have mostly been overlooked in the past. The presence of a coxal outline is therefore dependent on the degree of sclerotisation, in our studied species maximally visible in e.g. the pleopods of amphipods (Figure 7B, C), in a medium state in phyllocarids (Figure 7D, E), and in its minimum visible in just the sclerites, as in stomatopods or decapods (Figure 7A, E).

The entire structural complex around the sclerites, now identified as a modified coxa, should have evolved before the stem species of all modern Malacostraca, but neither ontogenetic nor palaeontological data are available to elucidate this problem. Hence, to reflect the question of the evolutionary origin of the coxal body of malacostracan thoracopods, no matter if the anterior eight or the pleopods, cannot be discussed here at present.

Another, though more indirect, evidence are the observed apodemes. These belong to the morphology of the coxae of the anterior thoracopods too and serve as attachment devices for musculature running into the limb [4,5]. Such apodemes are also present on some of the sclerites, and scleritic elements might even comprise several pieces forming a ring (e.g. in *Meganyctiphanes norveciga* (Sars, 1857); see [5], their figure 3A). Thus there are further similarities between the basal area of pleopod limbs and thoracopodal coxae.

However, we refrain from confirming earlier authors, who had applied the term "coxa" to proximal (= scleritic) structures on malacostracan pleopods [54,55]. Sclerites themselves do not represent the coxa (or are its remains), but are solely part of it, calcifications within the cuticle of the coxa. Only in some cases they are equivalent to the sclerotised region of the coxa, i.e. when the rest has become little sclerotised. These are apomorphic states of a particular taxon, but not in the sense of degeneration. In this respect it is also unclear if the "proximal segment" of the pleopods Ungerer & Wolff [27] identified in embryos of *Orchestia cavimana* represent the same structure recognised here as the coxa.

The limb stems of pleopods are generally elongated and tubular. Modification of their connection to the body by a de-sclerotisation of parts of the coxa and by parallel enhancement of rigidity by the calcitic sclerites led to the specific movements of the pleopods (independent if the presence of sclerites originates from development or retention). Initially leaving all four pivot joints, movement of the pleopods was mainly in antero-posterior direction. In connection to other life styles and modes of locomotion more changes occurred on the pleopods [56-59], although the specific functions of the basal joint have not yet been touched (but see [29]). Further investigations on this would therefore be very helpful, also including life studies of the pleopods of the according taxa.

## Conclusions and Outlook

Our study of scleritic elements in the proximal-most limb joint area of malacostracan pleopods does not only yield new data about this structural complex, but it also demonstrates its potential to enlighten us about phylogeny. Calcified sclerites are not simply present in the body–limb articulation of malacostracan pleopods, but also participate in the formation of joints with limb basipod and sternites medially and tergopleurae laterally by providing the corresponding depressions or knobs to those of the associated elements. Some of the sclerites have, in addition, internal apodemes for internal muscles to attach there. Number and position of the sclerites turned out to be taxon-specific and constant, even conserved in strongly modified pleopods. A higher number of sclerites (7 or 8) seem to be part of the ground pattern for Malacostraca.

Caridoida are characterised by a reduced number of sclerites, but retain all major components. Within Caridoida, the sister-group relationship of Euphausiacea and Decapoda is supported by the occurrence of a similarly reduced and specific pattern of sclerites; we interpret this as autapomorphic for Eucarida.

Within Peracarida the original sclerite pattern seems to have become simplified, with the appearance of a specific extension connecting the median sclerite and sternite in Amphipoda and Mysida points to a further specialisation. Independent evolution of Lophogastrida and Mysida is supported.

Furthermore, we consider these sclerites as calcitic components in the cuticle proximal to the basipod. In most cases, however, this coxa area is not a completely sclerotised ring element; sclerotised areas are mainly limited to the median and lateral edges – also more or less the sites of the calcitic sclerites – the posterior area is membranous.

Further studies on this character complex are demanded and promising: Particularly the functional morphology of the calcitic sclerites in their role to assist the joints and influence to movability of the pleopods in line with the role of the decalcified areas of the coxa should be examined in more detail. Further investigations of representatives of other in-group Peracarida, i.e. of the Mancoida would also be helpful in order to improve the resolution of the system within Peracarida.

In a wider array, the identification of a coxa on now all thoracopods of Malacostraca might also be interesting for the ongoing discussion about tracheate or insect relationships with crustaceans or even malacostracans and the problematic role of Remipedia. Relationships have been supposed in a whole suite of papers, also based on sclerotised elements present at the limb base in these taxa [1,60-65], which makes it even more challenging to compare more scleritic data between all these groups. Indeed closer examination of the situation in Remipedia and the Tracheata seems promising to us, not least due to the presence of scleritic elements in their limb joints.

## Methods

### Material

We examined 3–8 individuals of 16 species representing different malacostracan taxa (Figure 1, Table 4). *Allanaspides hickmani* (museum number: 26633) and *Paranaspides lacustris* (museum number: 26632) were from the Naturkundemuseum Berlin, Germany. *Nebaliopsis* sp. specimens were from the Senckenberg Naturmuseum Frankfurt, Germany (museum number: SMF-43552) and the Zoological Museum of Copenhagen, Denmark. All *Nebaliopsis* specimens were damaged due to capturing them in the deep sea; only their cephalothoracic shields and complete pleons were present, which was sufficient for our study.

We studied the body–limb articulation of the pleopods 1–5 in each species, except for the phyllocarid representatives, as pleopods 5 and 6 are small and reduced. The pleopods 6 are often modified into uropods and were therefore excluded here, as they were subject of another study [28]. Taxa without pleopods or with strongly reduced pleopods, i.e. Bathynellacea, Thermosbaenacea, and in-group Peracarida, as well as in-group Decapoda were omitted.

### Methods

*Regeneration. –* Specimens of *Gonodactylus chiragra* were available as dried samples. They were regenerated with a 5% NaCl solution (Table 4), in which the specimens were deposited [66] till the segments and appendages were movable again what took three days. The specimens of *Go. chiragra* were further processed for staining with Alizarin Red (Table 4).

*Staining with Alizarin Red.* – Alizarin Red tinges calcified parts of the cuticle purple while membranous areas remain white. This allowed detection of small, calcified areas ("sclerites") in the pleopod-body articulation. We followed the protocol given by Brösing et al. [67]: specimens (Table 4) were cooked at 100°C in a 10% KOH-solution for 1 h, which dissolves the inner organs while only the cuticle remains. A spatula point Alizarin Red was added and the specimens were cooked for further 15 minutes, during which the actual staining took place. Afterwards the specimens were washed with distilled water and stored in 70% ethanol for documentation. This was performed with representatives of several species (Table 4), but not all. The specimens loaned from different museums were neither stained with Alizarin Red nor dissected but examined with fluorescence microscopy (Table 4).

*Macrophotography.* – Most overview images of whole specimens were obtained with a Canon EOS 450D camera with an EFS 60 mm objective under crossed polarised filters [68-70]. The depth of field was improved by recording image stacks and combining the single images into one sharp photograph with the software Combine ZP [69]. The overview images of the other specimens (*Al. helonomus*, *Hyperia* sp., *Pa. lacustris*, and *Neo. semissis*; see Table 4) were documented with fluorescence microscopy and composite imaging [35,69].

Images of the pleopod-body articulations of Alizarin-Red stained specimens were either made with a Canon EOS 450D camera with a MP-E 65 mm objective on a light table to enhance the contrast, or by attaching the camera to a Zeiss Axioskop microscope photographing with transmitted light (Table 4, column AR). The depth of field was improved accordingly. All images were further processed in Adobe Photoshop CS3 resp. CS4.

*Fluorescence microscopy*. – Specimens borrowed from museums were not stained with Alizarin Red. To guarantee an equally distinct documentation, the pleopod-body articulations of these specimens (*Hyperia* sp., *Nebaliopsis* sp., *Neo. semissis*^*,*^*Pa. lacustris*, and *Al. hickmani*; Table 4) were documented via fluorescence microscopy ([34,70], Table 4). Calcified sclerites are fluorescent whereas the membranous parts are not. Thus, the sclerites are displayed bright and whitish in a fluorescence micrograph, whereas the membranous parts are comparably dark. The specimens were documented with a Zeiss Axioskop 2 with an Axiocam under UV light (356 nm).

*Scanning electron microscopy. –* Preparation and documentation followed the protocol given by [33].

*Dissection.* – Those specimens, which were stained with Alizarin Red and were not loaned from a museum, were further dissected with pointed forceps and fine scissors (Table 4). By that it was possible to isolate the sclerites and detect presence of apodemes, the internal attachment points of muscle fibres.

*Terminology.* – The terminology applied was taken mainly from Walossek [6,8] and collaborators [10,12]. The sclerites were numbered consecutively in each species. The various numbers do not intend inter-specific homologisation. For the ground-pattern reconstructions numbering was omitted, but the positions of the sclerites were used to specify them (detailed explanations for our schs. in Table 1, Table 2 and Table 3). In most cases only the left half of a pleomere was illustrated, and the sternitic wings omitted.

Remarks concerning the term "sternite": A sternite is here understood as the median, sclerotised area between the insertions of a pair of post-oral appendages [71]. This sclerotised area is part of the entire ventral region of a segment, the rest of the area is membranous. The sternite provides the median attachment point of the appendages, better their basipods, the outer attachment is between basipod and tergopleura, both enforcing an antero-posterior swing of the limb. In the well-sclerotised and calcified malacostracans the attachments are likewise improved and more rigid, in the simple form consisting of pivot joints, ball-and-socket joints that connect the coxa of thoracopods 1–8 with the sternite and tergopleura. Sternites may fuse with one another along the body, often within entire tagmata, but never in the pleon of Malacostraca. There modifications may occur in the form of extensions around the limbs anteriorly and posteriorly into so-called wings, sometimes even encompassing the complete insertion areas of the limbs. The two wings might even fuse laterally with the tergopleura, forming a rigid skeletal system around the pleopodal insertion areas, such as in a lobster. However the indications of the pivots are still present.

*Ground-pattern reconstruction. –* Mostly two species of one taxon were examined (Table 4). By comparison of the results and applying the parsimony principle we reconstructed a possible ground-pattern state for the taxa [72,73] For this we act on the assumption of monophyly of the examined taxa. Richter & Scholtz [43] demonstrated that our taxa most likely represent closely related forms. We produced small schs. to illustrate the distribution of calcified elements in the surrounding membranous region (of the coxa; see discussion) for each species group with a similar pattern (Table 1 and Table 2, Figure 6). Note that the actually three-dimensional conditions are projected on two dimensions.

*Phylogenetic analysis and mapping. –* We mapped our reconstructions by hand onto two recent, morphology-based phylogenies proposed for Malacostraca: those of Wirkner & Richter [36] and Tabacaru & Danielopol [35]. We also considered the phylogeny proposed by Kobusch [45,51] for peracaridan in-group relationships. After mapping a critical *a posteriori* evaluation of the character distribution using the parsimony principle was made, an approach well suitable for morphological phylogeny analyses [18,24,28,74-76] and described in detail in [74,75]. Such approach can also be regarded as a test of the relationships proposed in these phylogenetic trees [28,74-76].
